# Rational Design of Covalent Organic Frameworks for Enhanced Reticular Electrochemiluminescence and Biosensing Applications

**DOI:** 10.3390/bios15110760

**Published:** 2025-11-16

**Authors:** Bing Sun, Lin Cui

**Affiliations:** 1 School of Science, China University of Geosciences (Beijing), Beijing 100083, China; 2 College of Chemistry, Chemical Engineering and Materials Science, Shandong Normal University, Jinan 250014, China

**Keywords:** covalent organic frameworks, electrochemiluminescence, structural design, charge transfer, biosensors, environmental monitoring, food safety assay

## Abstract

Electrochemiluminescence (ECL) has evolved into a powerful analytical technique due to its ultra-high sensitivity, low background noise, and precise electrochemical control. The development of efficient ECL emitters is central to advancing this technology for practical applications. Covalent organic frameworks (COFs) have recently emerged as promising candidates for constructing high-performance ECL systems. The tunable porosity, ordered π-conjugated structures, and versatile modular functionalities of COFs provide fast massive transport, effective electron transfer, rapid interfacial electrochemical reaction, and enhanced ECL emission performance. This review provides a comprehensive overview of the rational design strategies and structural engineering for COF-based ECL materials at the molecular level. Linkage chemistry, monomer selection (luminophores and π-conjugated non-ECL motifs), precise framework regulation, post-synthetic modification, composite formation, and other ECL enhancement strategies were discussed for developing COF-based ECL emitter. Both the incorporation of aggregation-induced emission and intramolecular charge transfer mechanisms are included to enhance ECL efficiency. Donor–acceptor conjugation, heteroatom element content, isomerism, substitution, and dimensional direction were regarded as effective strategies to regulate the electronic structure and band diagrams for designing high-performance ECL systems. The role of COFs as both active emitters and functional scaffolds for signal amplification is critically examined. Furthermore, their diverse analytical applications across biosensing, food safety, environmental monitoring, and chiral recognition are highlighted. By correlating structural features with ECL performance, this review offers insights into the design principles of next-generation reticular ECL materials and outlines future directions for their practical deployment in sensitive and selective sensing platforms.

## 1. Introduction

Electrochemiluminescence (ECL), also termed electro-generated chemiluminescence, is a light-emitting phenomenon that originates from high-energy electron-transfer (ET) events between radical intermediates electrochemically produced at an electrode–electrolyte interface [1,2]. ECL has emerged as a powerful analytical technique that combines the advantages of electrochemical control and optical detection, offering ultra-high sensitivity, low background noise, and excellent temporal resolution [3]. Unlike conventional photoluminescence, ECL does not require an external light source, thereby minimizing interference from scattering and autofluorescence and enabling robust operation in optically dense matrices such as whole blood, environmental effluents, or complex food extracts [4]. The analytical signal is directly proportional to the concentration of the luminophore or its co-reactant, providing a wide linear dynamic range together with excellent temporal and spatial control imparted by the applied potential. These properties have cemented ECL as the quantitative foundation underpinning commercial analyzers and have spurred a rapidly growing body of research in analytical applications [5,6,7].

Fully realizing the analytical potential of ECL is contingent upon the rational design of luminophores that integrate high photoluminescence quantum yields, reversible redox behavior, efficient charge transfer, and electrochemical excitation at the electrode interface [8,9]. Therefore, the deliberate development of ECL-active materials with tailored electronic properties, enhanced stability, and superior charge transport capabilities is crucial for improving the performance of ECL-based sensors. The typical inorganic ECL emitters such as [Ru(bpy)_3_]^2+^ and CsPbBr_3_ perovskite nanocrystals exhibit high quantum yields in aqueous media [1,10], but suffer from limited post-modification flexibility and stable immobilization on electrodes due to high water solubility. In contrast, metal-free organic aggregation-induced ECL (AIECL) emitters, such as tetraphenylethene (TPE) and its derivatives, offer tunable properties and represent an innovative approach to ECL generation in aqueous media [11]. However, these AIECL systems often lead to random and disordered structures due to their reliance on the aggregation through restricted molecular rotation in poor solvents, which hinders the rational design of a diversified luminophore family through controlled structural, compositional, or spatial modulation, limiting their adaptability to evolving detection requirements. The AIECL also indicates the role of solid-state emitters in reducing the non-radiative relaxation and enhancing the electrocatalytic effect for brilliant ECL emission [12,13]. Meanwhile, various porous materials such as metal–organic frameworks (MOFs) and covalent organic frameworks (COFs) attracted much attention as emerging reticular ECL emitters, owing to their high functional and structural tailorability, substantial capacity for hosting active sites, and enhanced activity attributable to nanoconfinement effects [14,15].

Covalent organic frameworks (COFs), a class of crystalline porous polymers with predictable structures and tunable functionalities, have recently gained significant attention as next-generation platforms for ECL applications [16]. Built from light elements and linked by strong covalent bonds, COFs offer exceptional chemical stability, large surface areas, and long-range π-conjugation, which are favorable for facilitating both mass and electron transfer [17]. Benefiting from the cationic or hydrophobic nanochannels preconcentrate co-reactants, improved electron transfer and framework rigidity suppresses self-quenching, COF-based ECL systems demonstrate brilliant emission and enhanced luminescence efficiency. In recent years, significant progress has been made in developing predictable COF-based ECL materials through structural design strategies such as monomer engineering, linkage optimization, pore size precise control, post-synthetic modification, and composite formation. Incorporation of donor–acceptor (D–A) units, aggregation-induced emission (AIE) motifs, and redox-active sites directly into the framework has been shown to effectively modulate intrareticular charge transfer (IRCT) or intramolecular charge transfer (ICT), suppress non-radiative decay, and promote co-reactant enrichment, thereby significantly enhancing ECL efficiency [18,19,20,21]. The higher specific surface area and abundant modification sites of COFs endow them as good scaffolds to load luminophores via adsorption, coordination, host-guest interactions, and covalent graft as well as to facilitate the access of co-reactants or improve the charge transfer [22]. Beyond structural design, COFs have also shown great promise in a wide range of analytical applications. Their high porosity and surface functionality facilitate the immobilization of recognition elements such as antibodies, aptamers, and molecularly imprinted polymers, enabling selective detection of target analytes [14]. As a result, COF-based ECL sensors have been successfully applied in food safety analysis (e.g., detection of antibiotics and toxins), environmental monitoring (e.g., heavy metal ions and organic pollutants), biomedical diagnostics (e.g., cancer biomarkers and pathogens), and even chiral sensing, highlighting the versatility and adaptability of COFs in addressing diverse analytical challenges [23,24].

**Figure 1:**
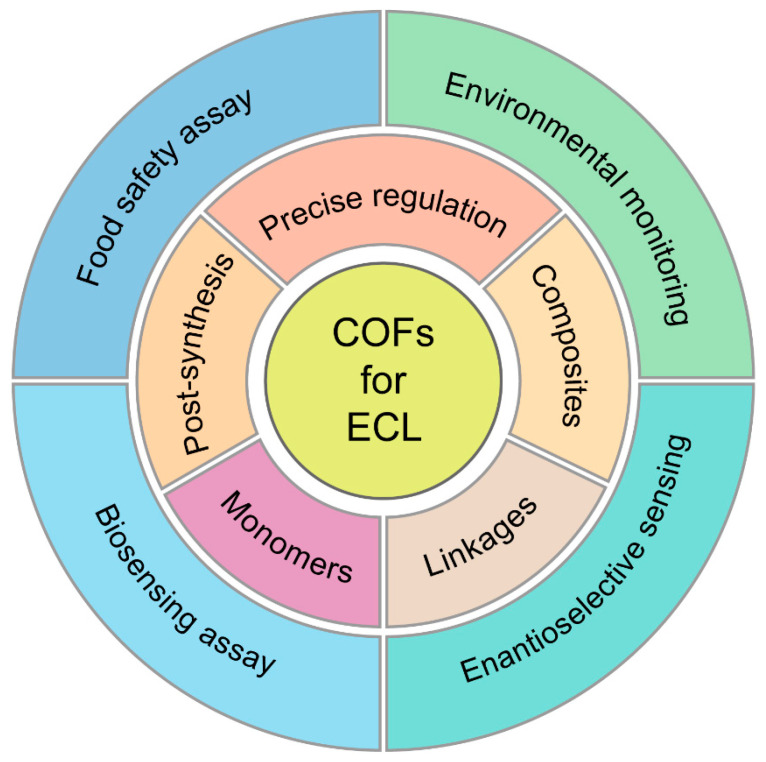
Schematic diagram of the design and applications of COFs with enhanced ECL performance.

Since the most advances of COF-based ECL systems and key studies published after 2020, some reviews about the applications of COFs in ECL have already been published [7,14,16,23,24,25,26,27,28]. Many reviews focus on the fabrication of COF-based ECL systems for sensitive sensing along with COF-based electrochemical/fluorescent sensors. COF-based ECL systems are only summarized as a part compared to multiple porous materials. In this review, we critically survey the evolving design toolbox for reticular ECL materials, moving from the choice of luminescent or π-conjugated non-emissive monomers, through linkage chemistry and down to angstrom-level framework editing (such as element stoichiometry, constitutional isomerism, regio-substitution and dimensionality, etc.). Advanced post-synthetic metalation, covalent functionalization, and hybridization with conductive or catalytic guests are evaluated alongside photophysical strategies that exploit aggregation-induced emission and intramolecular charge transfer to maximize excited-state yields. We delineate how COFs operate simultaneously as primary emitters and as signal-amplifying scaffolds, and further benchmark their utility across biosensing assays, food-safety screening, environmental surveillance, and chiral discrimination. The main line of this review is highlighted in Figure 1. By mapping structure–property relationships onto analytical figures of merit, the review identifies transferable design rules for next-generation COF-based ECL platforms and highlights the remaining challenges that must be overcome to translate these designer frameworks into deployable sensors.

## 2. Rational Design of COFs for Enhanced ECL

**Figure 2:**
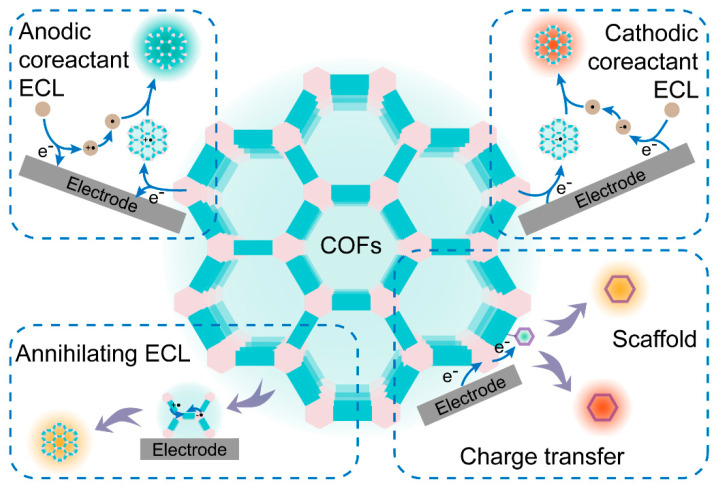
Schematic diagram of ECL mechanisms based on COFs. The brown ball represents the co-reactant added to the ECL system. ECL involves redox reactions at the electrode to generate radical ions of the COF-based emitters and/or co-reactants (such as TPrA, S_2_O_8_^2−^, H_2_O_2_, O_2_, etc.) at anodic or cathodic electrodes. These species react to form an excited state, and further generate anodic ECL (left-top route) and cathodic ECL (right-top route), respectively. Direct annihilation of the formed radical species in the COF skeletons also generates bright ECL emission (left-bottom route). Additionally, COFs can serve as the scaffolds to immobilize ECL emitters or act as charge transfer media for enhancing the ECL emission (right-bottom route). The narrow blue arrows indicate the electron transfer directions and the purple arrows represent the reaction procedures (left-bottom route) or classification (right-bottom route).

Efficient ECL is governed by the kinetics and thermodynamics of radical-mediated charge transfer between an emitter and its co-reactant. In reticular emitters, the ECL efficiency is determined by IRCT-mediated radical annihilation. COFs offer atomistic control over this process by stabilizing radicals at predefined catalytic nodes while simultaneously shortening electron-transport pathways to promote exciton generation for ECL emission [20,29]. The radical lifetimes must be balanced: overly labile intermediates decompose before productive electron exchange, whereas excessively stabilized species trap charge and suppress exciton formation. High-energy co-reactants such as tripropylamine (TPrA) and peroxysulfate (S_2_O_8_^2−^) are usually employed to generate intense ECL emission at anodic or cathodic electrodes. Green alternatives (such as dissolved O_2_ in aqueous solutions) are further developed to avoid the introduced corrosion, biotoxicity, and matrix interference of these exogenous co-reactants [30]. The typical ECL mechanisms based on COFs are summarized in Figure 2 according to recent advances in progress.

The predetermined chemical structures, specific functionalities, and tunable porous properties constitute the principal design metrics for the rational fabrication of high-performance COF-based ECL emission. Direct polymerization of intrinsically luminophore monomers with crystalline order and permanent porosity is an effective way to construct enhanced ECL-active COFs by suppressing the aggregation-induced non-radiation relaxation and quenched emission. Remarkably, “silent” building blocks can be rendered emissive by embedding spatially separated donor–acceptor pairs that enable through-bond or through-space charge transfer; directional linkages and extended π-conjugation amplify this effect. Halogenation, dimensional control (1D vs. 2D), and post-synthetic metalation provide additional handles to fine-tune radical stability, charge mobility, and photon yield. Post-synthesis of preformed COFs and composite formation also serve as scaffolds to immobilize ECL luminophores or facilitate the charge transfer for strong ECL emission [31]. The scaffolds with high specific surface area expose more metal-/hetero-atom sites for surface-mediated S_2_O_8_^2−^ decomposition, reduce diffusion barriers, and accelerate electron shuttling, thereby boosting cathodic ECL. Molecular-level engineering of COFs by tuning building blocks, linkage chemistry, precise structure, post-synthesis, and composite materials is discussed in this part for permitting predictive regulation of radical dynamics and establishing a clear structure–property roadmap for next-generation, green ECL sensors.

### 2.1. Linkage Chemistry

**Figure 3:**
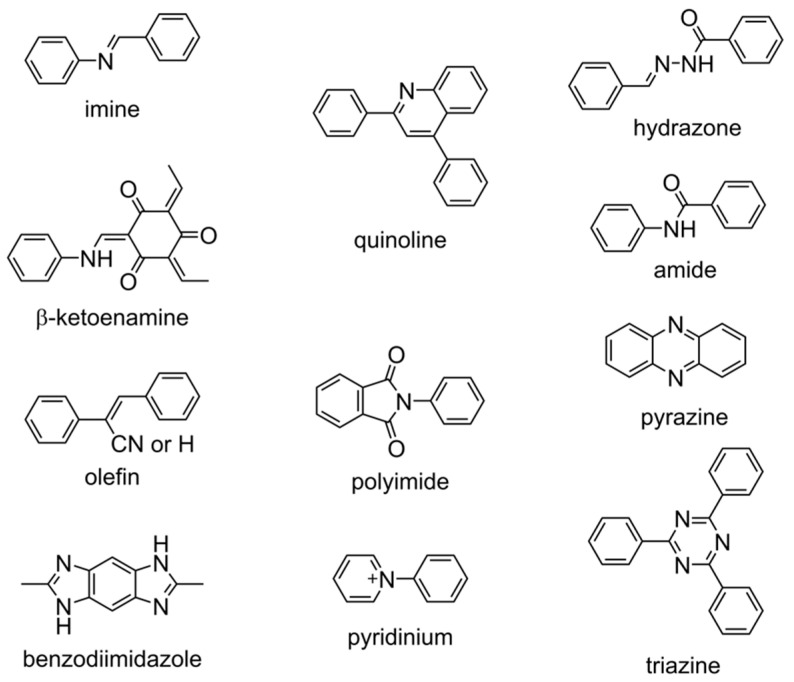
Typical linkages of COF lumiphores with enhanced ECL emission.

Linkages forged through diverse condensation chemistries, such as Schiff base, Knoevenagel, aldol, Zincke, and related protocols, provide robust covalent connectivity between monomers, enabling the construction of functionalized covalent organic frameworks for reticular electrochemiluminescence. Beyond structural integrity, the degree of π-conjugation, intrinsic conductivity, and dipole moment inherent to each linkage critically govern IRCT, radiative relaxation dynamics, and overall ECL efficiency. Representative bonding motifs exploited in COF-based ECL materials are collated in Figure 3. Among the repertoire of linkages, imine remains the most frequently exploited motif for COF construction; however, the intrinsic polarization of the C=N bond can severely suppress ECL by accelerating non-radiative decay pathways. As imine-derived motifs, rigid and planar β-ketoenamine linkages extend π-conjugation across an atomically flat scaffold, intensifying intramolecular charge transfer while simultaneously suppressing non-radiative decay, thereby boosting ECL efficiency [32]. Moreover, Post-synthetic cyclisation of imine bonds into fused quinoline rings rigidifies the backbone, extends π-delocalization, and quenches vibrational relaxation, thereby activating intense ECL [33].

Olefinic or alkenyl motifs represent a class of essential linkages that are generated via Knoevenagel or aldol condensation of electron-deficient triazines/cyanurates with aldehyde-functionalized donors, replacing polar, rotationally labile C=N bonds with fully sp^2^-hybridized C=C bridges [17]. This architectural swap rigidifies the backbone, suppresses phenyl-ring torsion, and establishes uninterrupted π-delocalization that shortens electron/hole migration paths and narrows the band gap. The resulting anion radicals, stabilized by electron-withdrawing acrylonitrile segments, undergo accelerated, radical-stability-dependent recombination, while the complete exclusion of nitrogen-mediated quenching sites restores high photoluminescence efficiency. Olefin-linked D-A COFs exhibit intense, color-tunable ECL that far surpasses their imine- or hydrazone-connected counterparts, underscoring C=C bonds as pivotal design elements for next-generation, low-overpotential reticular emitters. Furthermore, a volcano plot linking ECL intensity to anion-radical lifetime is revealed in nitrile-functionalized pyrene-based COFs by installing a graded series of nitrile acceptors [34]. CN-COF-2, bearing one nitrile per linker, affords optimal radical stabilization and 78-fold brighter emission than either under- or over-acceptor analogues, establishing radical lifetime as a tunable descriptor for metal-free reticular ECL.

Other linkages included hydrazone, aminal, pyrazine, polyimide, benzodiimidazole, and triazine. Particularly, the pyrazine-linked COF features an atomically flat, fully π-conjugated backbone that delivers exceptional electrical conductivity; in synergy with its high porosity and pre-electrolysis activation, this yields intense ECL [35]. A crystalline, aminal-linked COF formed via condensation of a carboxaldehyde with piperazine also exhibits outstanding electrochemiluminescence when paired with appropriate co-reactants [36]. Covalent triazine frameworks (CTF) demonstrate good crystallinity and stability and usually serve as the scaffolds to anchor ECL emitters (such as Ru^2+^) [37,38]. Additionally, ionic COFs have also been designed and synthesized via the Zincke reaction, establishing a new class of ECL luminophores [39].

### 2.2. Luminophore Monomers

**Figure 4:**
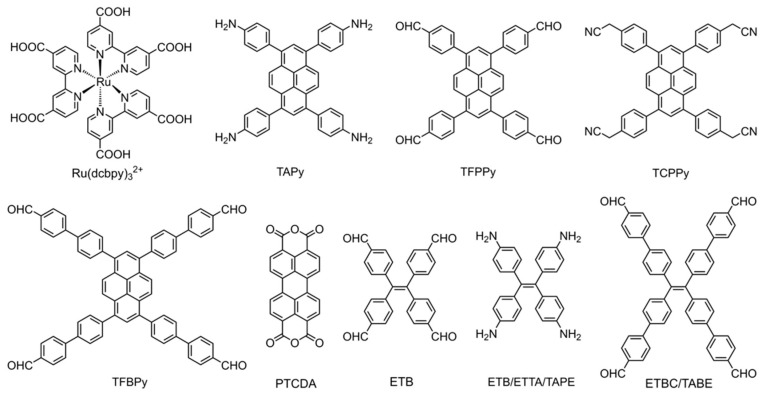
Reported luminophore monomers in previous works for fabricating COF-based ECL emitters.

Incorporating ECL emitters into reticular frameworks is a direct and effective way to fabricate high-performance ECL-active COF materials. Typical luminophore monomers for fabricating COF-based ECL emitters are summarized in Figure 4. Ru-complexes are regarded as an extensively studied luminophore for brilliant ECL output. A highly stable Ru-COF has been strategically constructed as an ultrasensitive ECL probe from the classic luminophore tris(4,4′-dicarboxylicacid-2,2′-bipyridyl)ruthenium(II) (Ru(dcbpy)_3_^2+^) and benzene-1,2,4,5-tetramine (BTA) with benzobiimidazole linkage, yielding a crystalline architecture in which the luminophore is covalently stitched into the skeleton rather than physically entrained [40]. The ordered topological structure of Ru-COF maximizes the volumetric density of Ru(dcbpy)_3_^2+^ centers with homogeneous distribution, provides open channels for the transport of electron/ion and co-reactant fluxes, and facilitates the electrochemical excitation of Ru(dcbpy)_3_^2+^ units for excellent ECL emission. The covalently binding Ru(dcbpy)_3_^2+^ in Ru-COF demonstrates high water stability, suppressing the leakage of Ru-complexes for enhanced ECL stability. The integration of high luminophore loading, efficient charge–mass transport, and superior hydrolytic stability positions Ru-MCOF as a good ECL emitter for ultrasensitive assays.

Metal-free organic luminophore building blocks are also developed for constructing high-performance ECL-active COFs. Pyrene-based luminophore monomers are explored to fabricate enhanced ECL-active COFs (as shown in Figure 4). For instance, TFPPy-DMeTHz-COF constructed from 1,3,6,8-tetrakis(4-formylphenyl)pyrene (TFPPy) and 2,5-dimethoxyterephthalohydrazide (DMeTHz), demonstrated surface-state-governed ECL emission rather than band-gap recombination process [41]. Its ECL performance is independent of dissolved O_2_ or its radical offspring (O_2_^•−^ and •OH). Instead, hydroxide ions in the electrolyte act as the dominant co-reactant, affording a pH-linear response (pH 3–10) with a relative ECL efficiency of 21.7%. When TFPPy is alternately spaced with (1,1′:3′,1″-terphenyl)-4,4″-diamine (TPh), the resulting TFPPy-TPh-COF suppresses aggregation-caused quenching (ACQ) by locking the pyrene units into a rigid, periodically dilated lattice [42]. Concomitant restriction of intramolecular rotations minimizes non-radiative losses, delivering ECL outputs that surpass those of neat TFPPy aggregates and enabling sensitive detection of malathion. In addition, perylene-based monomers are also employed for constructing ECL-active COFs. Li et al. [43] reported a robust PTCA-COF with stable and intense ECL fabricated from perylene-3,4,9,10-tetracarboxylic dianhydride (PTCDA) with melamine (MA). The crystalline lattice enforces a coplanar, π-stacked array of PTCDA fluorophores, while sub-nanometer pores impose spatial confinement that suppresses non-radiative relaxation; together, these effects yield a stable and brightly emissive COF without external luminophore doping.

To overcome the restriction of the ACQ effect in COFs integrated from organic luminophores, AIECL luminophores represent promising building blocks for engineering high-performance COF-based ECL emitters [18,44]. COFs that lock AIE luminogens into a rigid, π-delocalized lattice give up to two orders of magnitude brighter ECL than the corresponding molecular aggregates, even in the absence of exogenous co-reactants. TPE and its derivatives are extensively used as AIEgens and employed in developing strong AIECL materials. Luo et al. [45] reported that incorporating the AIE monomer 4,4′,4″,4‴-(ethene-1,1,2,2-tetrayl)tetrabenzaldehyde (ETB) into a sp^2^-carbon-linked COF produces framework-induced electrochemiluminescence (FIECL) that is ~100-fold brighter without any exogenous co-reactant than aqueous aggregates of the free monomer. Long-range π-order backbone locks ETB rotors in place, suppressing torsional relaxation of the chromophore and non-radiative decay, thereby accelerating intra-framework charge transport. Density-functional theory (DFT) confirms a lowered energy band relative to the monomer, accelerating electron transfer within frameworks for the rapid formation of the emissive excited state and stronger FIECL emission. Furthermore, a conceptually related “covalent rigidification-triggered ECL (CRT-ECL)” is realized in ultrathin tetra-(4-aldehyde-(1,1-biphenyl))ethylene-piperazine COF (TABE-PZ-CON) [46]. Covalent immobilization of massive TABE luminogens within the rigid lattice suppresses internal rotation, while the nanometer-thin porous sheets shorten ion/electron/co-reactant diffusion paths. Both effects increase the number of electrochemically addressable emitters and boost overall ECL efficiency.

### 2.3. Precise Regulation of ECL-Active COFs

**Figure 5:**
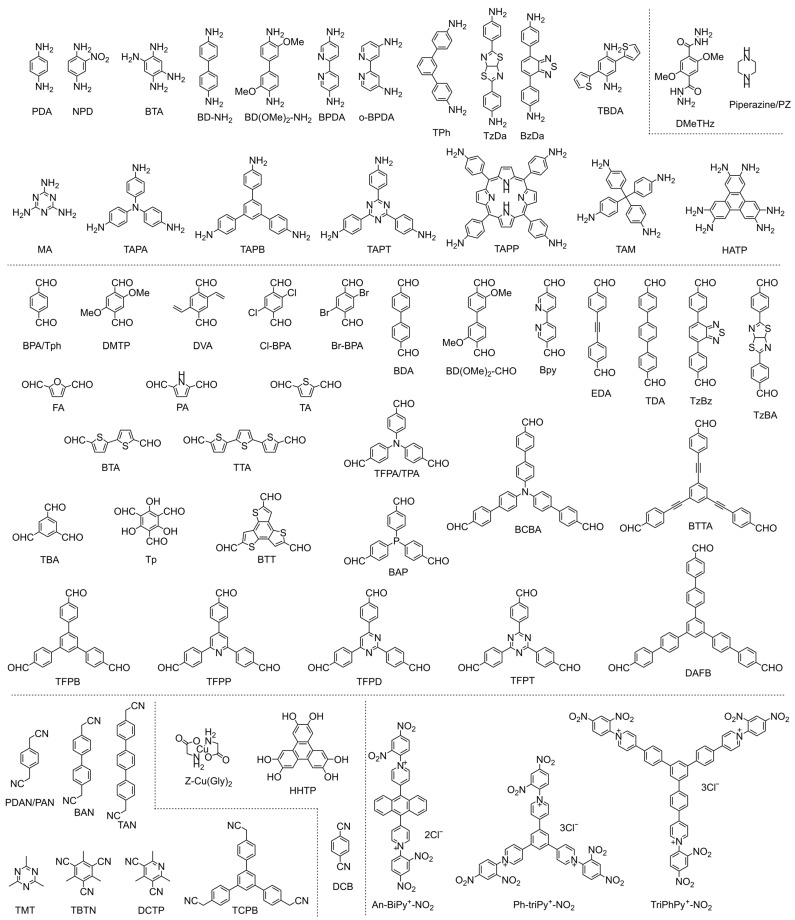
Reported amine, piperazine, hydrazine, aldehyde, and other monomers in previous works for fabricating ECL-active COFs.

Recent breakthroughs in COF-based ECL have established that judicious manipulation of π-conjugation and band-gap topology can transform ostensibly “dark” monomers into high-performance emitters. Typical monomers for fabricating COF-based ECL emitters are summarized in Figure 5. The following discussion delineates how donor–acceptor pairing, heteroatom engineering, constitutional isomerism, regio-substitution, and dimensional control act in concert to activate and amplify ECL within reticular architectures.

#### 2.3.1. Donor–Acceptor π-Conjugation and Bandgap Regulation

**Figure 6:**
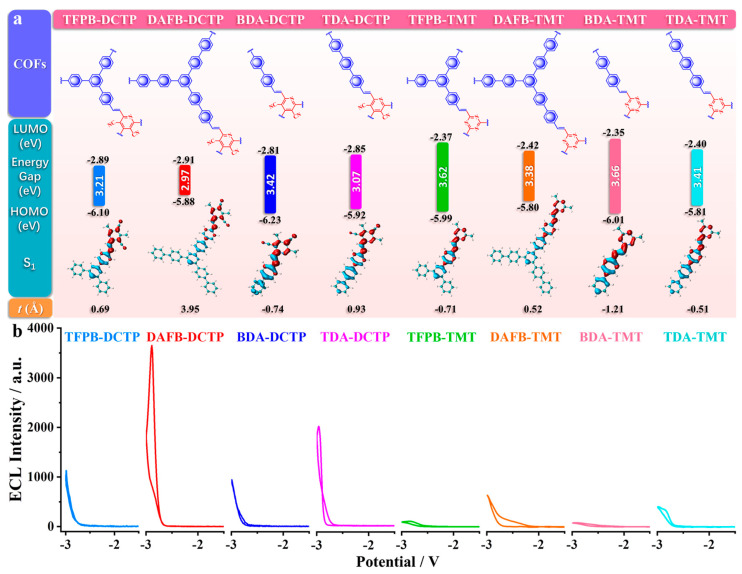
Effect of the extended D-A conjugation length of COFs on ECL performance. (**a**) Building blocks for COF construction and calculated HOMOs (blue), LUMOs (red) and band gaps corresponding to COFs. (**b**) Comparison of ECL intensities of COFs in 0.1 M phosphate buffer solution with 0.1 M KCl between 0 V and −3.0 V. PMT: 800 V, pH 7.5, potential scan rate: 100 mV s^−1^. Reproduced under terms of the CC-BY license [17]. Copyright 2021, Ya-Jie Li et al., published by Springer Nature.

Precise modulation of D-A stoichiometry and π-topology serves as a primary lever for band-gap engineering in COFs, simultaneously narrowing the electronic gap and shortening the electron-hole migration path. This synergistic lattice design accelerates exciton generation, spatial separation, and radiative recombination, delivering a versatile and predictable platform for high-performance ECL. Qiu and co-workers have advanced a versatile blueprint for amplifying the ECL of olefin-linked 2D D-A COFs that are intrinsically ECL-silent [17]. By systematically varying the D-A stoichiometry, they generated COFs that harvest dissolved O_2_ as the sole oxidant in aqueous electrolyte, eliminating the need for exogenous co-reactants while affording intense ECL outputs. Reticular engineering of D-A pairs simultaneously stabilizes radical intermediates, integrates luminophore and co-reactant motifs to minimize the spatial separation between anion/cation radicals, and deploys conjugated linkers to extend π-delocalization, thereby accelerating inter-radical charge transport. From the electronic view, periodic D-A alternation in COFs localizes the lowest unoccupied molecular orbitals (LUMOs) or conductance bands (CB) on acceptor columns and the highest occupied molecular orbitals (HOMOs) or valence bands (VB) on donor columns, creating an ICT manifold that efficiently separates and subsequently recombines charge under electrochemical excitation. The extended D-A lattice narrows the bandgap and compresses the electron migration, enabling efficient generation, separation, and radiative collision of electron-hole pairs and establishing a versatile COF platform for high-performance ECL (Figure 6).

**Figure 7:**
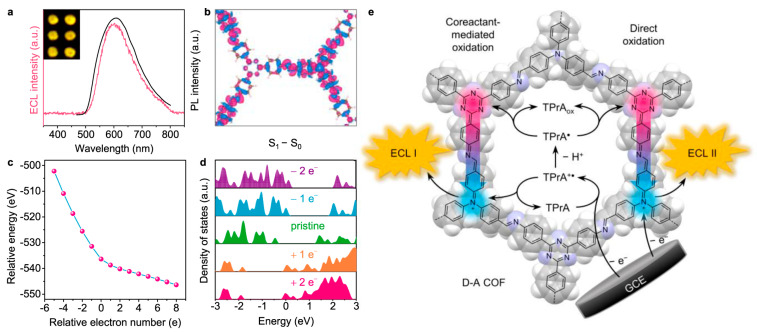
ECL performance of the D-A imine-linked COF based on triphenylamine donors and triazine acceptors and its Competitive oxidation ECL mechanism. (**a**) ECL at +1.40 V in 100 mM TPrA solution and PL spectra of D-A COF. (**b**) Electron density gain (blue) and loss (red) of D-A COF between 1st excited state (S_1_) and ground state (S_0_). (**c**) Relative energy and (**d**) density of states of D-A COF doped with different electron numbers. (**e**) Schematic illustration of ECL I and II generation mechanism via IRCT. Reproduced under terms of the CC-BY license [20]. Copyright 2021, Rengan Luo et al., published by Springer Nature.

Precise modulation of the D-A constituents serves as a primary lever for amplifying the ECL output of COFs by facilitating the charge separation and reduction in band gaps. Luo et al. [20] reported that a D-A COF comprising triphenylamine donors and triazine acceptors is engineered to harness tunable IRCT for ECL (Figure 7). The reticular D-A arrangement amplifies ECL intensity 123-fold relative to a benzene-bridged analogue with minimal D-A contrast, underscoring the pivotal role of spatially separated HOMO-LUMO distributions. Crystallinity- and protonation-dependent ECL responses corroborate that exciton generation is gated by IRCT efficiency. Moreover, an IRCT-mediated competitive oxidation pathway (co-reactant-assisted oxidation at low overpotential versus direct COF oxidation at high overpotential) produces distinctive dual-peak ECL profiles, offering a versatile route to ratiometric or multiplexed signaling. Cui et al. [47] proposed the stable D-π-A sequences directly into the COF backbones to serve as an internal ECL engine where the D → A vector accelerates intramolecular charge transfer through a fully π-conjugated olefin linkage highway that sustains ballistic charge transport along the lattice. Substituting the donor unit (4-[4-[4-(4-formylphenyl)-N-[4-(4-formylphenyl)phenyl]anilino]phenyl]benzaldehyde (BCBA) or 4-[4-[3,5-bis[4-(4-formylphenyl)phenyl]phenyl]phenyl]benzoic acid (DAFB)) alters the band gap at will, affording predictable control over emission intensity and wavelength. When the acceptor is fixed as 2,4,6-trimethylbenzene-1,3,5-tricarbonitrile (TBTN), the resulting COFs harvest dissolved O_2_ as the sole radical source. In pure water, the BCBA-TBTN COF attains an ECL efficiency of 63.7%, demonstrating that rational donor engineering alone is sufficient to activate and amplify framework-based ECL under environmentally benign conditions.

The ECL emission intensity and wavelength are also strongly correlated with the extended π-conjugation length of COFs. Twisting the biphenyl core while extending the π-scaffold emerges as a dual prerequisite for intense COF ECL: torsional strain suppresses rotational deactivation, and enlarged polyaromatic π-segment domains progressively compress the HOMO-LUMO gap. Substituting C–C single bonds with acetylene bridges rigidifies the backbone, deepens π-conjugation, and positions the alkyne moiety as an adsorption site that shortens the diffusion path to co-reactants [17]. These electronic and geometric modifications collectively accelerate charge separation, inhibit recombination, and enable systematic tuning of both emission wavelength and intensity across the visible region.

#### 2.3.2. Heteroatom Content Modulation

**Figure 8:**
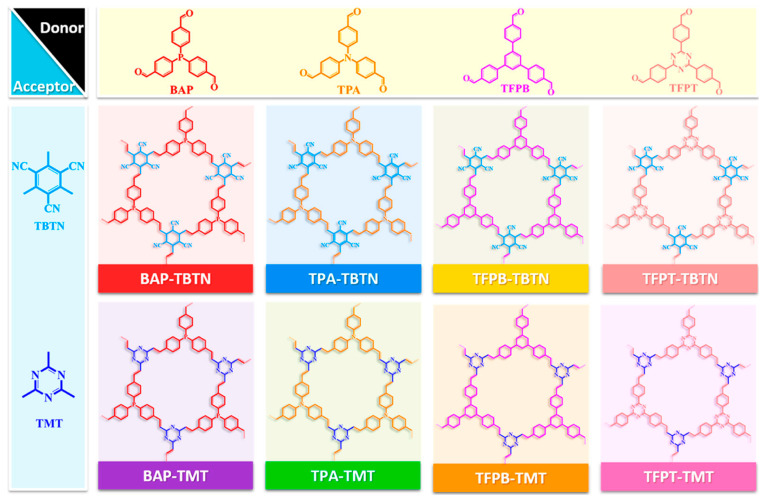
Olefin-linked D-A COF structures based on acceptor monomer (TBTN or TMT) and fine-tuned heteroatom-contented donor building blocks. Reproduced with permission from [48]. Copyright 2021 American Chemical Society.

**Figure 9:**
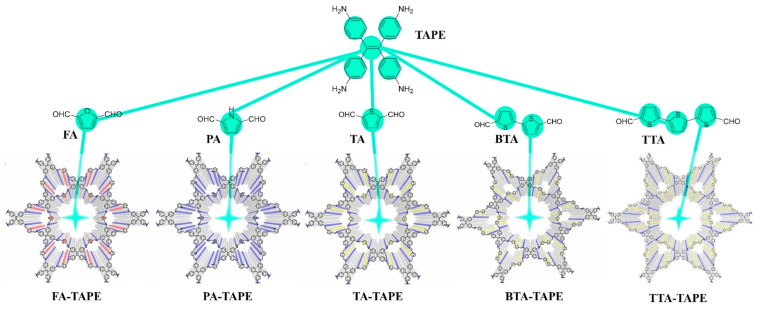
Synthetic diagrams of TAPE-based AIECL COFs with different aldehyde monomers. Reproduced with permission from [30]. Copyright 2025 Wiley-VCH.

Strategic incorporation and fine-tuning of heteroatoms within the building blocks emerge as a powerful paradigm for establishing D-A pairs and modulating the bandgap of COF, thereby affording significant ECL amplification. Li et al. [48] found that the BAP-TBTN COF constructed from electron-donating benzaldehyde-4,4′4″-phosphinidynetris (BAP), and electron-deficient TBTN demonstrated the strongest ECL emission compared to TPA-TBTN, BAP-TMT, and TPA-TMT D-A structures (as shown in Figure 8). Phosphorus incorporation endows the framework with strong electron-donating character, enabling direct D-A orbital overlap that instantaneously generates an IRCT state and drives highly efficient electrochemiluminescence. Hou et al. [30] proposed the band-gap engineering with *C*_4_-symmetric TPE and *C*_2_-symmetric five-membered heteroaromatic linkers for intense ECL-active COFs (Figure 9). The AIECL intensity of COF-based emitters increased by replacing the furan unit with 1H-pyrrole and further enhanced in the case of thiophene-based COF, which is attributed to the raised HOMO density along the entire twisted backbone and promoted π–π stacking. Moreover, progressive enrichment of the backbone with thiophene units raises the π-electron density, monotonically lowering the LUMO energy and compressing the band gap. The down-shifted LUMO promotes electron capture from co-reactant radicals, while the narrowed gap accelerates carrier mobility for amplifying ECL. TTA-TAPE COFs deliver 206 times higher ECL than their furan analogues and reach a ECL quantum efficiency of 639% (Ru(bpy)_3_^2+^/TPrA standard). High crystallinity preserves an ordered π-framework that sustains long-range charge transport and stabilizes radical cations/anions. Coincident with increased thiophene content, the specific surface area decreases and interlayer dislocations emerge; these periodic π-stacked faults create additional inter-sheet hopping channels that further shorten carrier-diffusion lengths, maximizing photon output.

Site-specific substitution of heteroatoms within the acceptor unit affords direct, angstrom-level control over its electron affinity, thereby amplifying intramolecular charge transfer across the D-A interface and markedly boosting ECL efficiency. Cui et al. [49] realized high-performance ECL from D-A-type BTT-COFs by polymerizing the strongly electron-rich 1,3,5-tris(4-formylphenyl)benzothiadiazole (BTT) core with a series of progressively electron-deficient olefinic acceptors. The continuous, columnar alternation of donors and acceptors rather than the statistically stacked aggregates that favor charge-carrier annihilation. The reticular architecture suppresses detrimental π–π stacking, immobilizes the D-A interface, and establishes a delocalized π-network in which the HOMO is confined to the BTT vertex while the LUMO is predominantly localized on the acceptor column. Increasing acceptor electron-withdrawing strength systematically narrows the band gap, shortens the valence-to-conduction transition distance, and accelerates exciton formation. Luo et al. [50] reported that systematic augmentation of framework nitrogen progressively lowers both reduction potential and emission energy of TBTN-N_x_-COFs (x = 0–3). Co-condensing TBTN with aldehydes whose aryl cores contain zero to three nitrogen atoms (TFPB, TFPP, TFPD, TFPT) yields isoreticular COFs in which each additional N-atom injects lone-pair electron density into the π*-manifold via σ*(N)–π*(aryl) hyperconjugation, monotonically depressing the LUMO and narrowing the band gap. The nitrogen-rich COFs thus function as tunable, low-overpotential ECL emitters whose luminescence potential and wavelength can be rationally programmed through heteroatom content.

#### 2.3.3. Structural Isomerism

**Figure 10:**
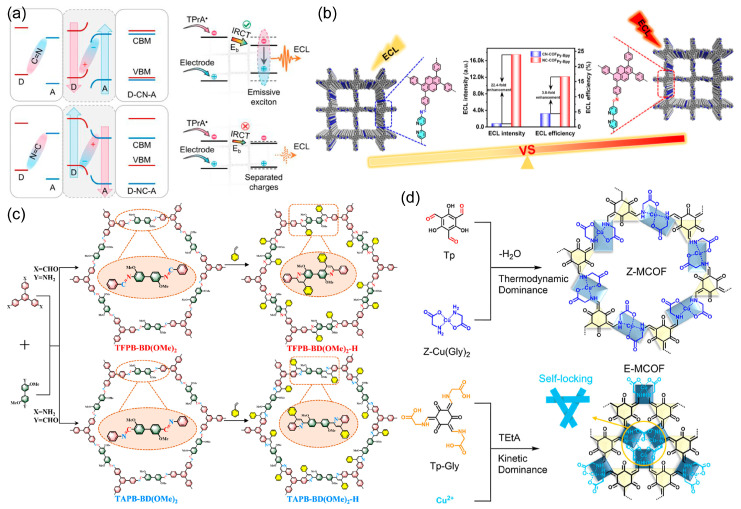
(**a**) Reticular ratchet effect based on the orientation of imine linkage for facilitating the charge transfer between D-A units. Reproduced with permission from [29]. Copyright 2024 American Chemical Society. (**b**) Schematic diagram of the effect of imine-linkage direction on COF ECL emission. Reproduced with permission from [51]. Copyright 2025 Elsevier. (**c**) Synthesis of isomeric imine-linked COFs and post-fused quinoline COFs. Reproduced with permission from [33]. Copyright 2023 American Chemical Society (**d**) Synthetic representation of cis-trans isomeric MCOFs (Z-MOF and E-MOF). Reproduced with permission from [52]. Copyright 2024 American Chemical Society.

The precise identity and geometric orientation of the linkage within a covalent organic framework constitute a critical design lever: they dictate the extent of π-conjugation, enforce or relax skeletal planarity, and modulate chemical stability. Even isomeric imine bonds, differing only in their directional connectivity, can impose distinct torsional profiles that translate into markedly disparate photophysical behaviors. Luo et al. [29] introduced the concept of “reticular ratchets,” wherein the directional alignment of D-A IRCT with imine-linkage polarization is exploited to govern charge-flow orientation and amplify ECL efficiency within COFs (Figure 10a). By converting isotropic charge diffusion into a directional ratchet, exciton formation can be dramatically amplified. Imine-linked COFs serve as programmable ratchets: aligning the D → A vector with the intrinsic N → C dipole of the CH=N bond accelerates electron flow from the N-terminus toward the C-terminus, whereas reversing the D–A orientation inverts band bending (C-end up, N-end down) and suppresses transport. Femtosecond transient absorption reveals that this dipole-imposed bias shortens electron/hole decay lifetimes and lowers exciton-binding energy, producing a 680-fold ECL enhancement as D–A contrast increases. Larger band-gap D-CN-A lattices exhibit higher exciton-binding energies than their D-NC-A counterparts, confirming that directional IRCT along the ratchet pathway efficiently funnels electrical and chemical energy into radiative recombination. Cao et al. [51] also engineered two isomeric imine COFs (NC-COF_Py-Bpy_ and CN-COF_Py-Bpy_) to probe the imine-bond orientation-governed ECL. In NC-COF_Py-Bpy_, the N → C dipole of each –CH=N– linkage is antiparallel to the donor (pyrene) → acceptor (bipyridine) charge-transfer axis, whereas CN-COF_Py-Bpy_ presents a parallel alignment. Counter-intuitively, the “opposed” NC-COF_Py-Bpy_ exhibits 22.4-fold higher ECL intensity and superior low-potential stability (TPrA co-reactant) than its “matched” counterpart (Figure 10b). Electronic structure analysis and kinetic modelling reveal that the reversed imine direction enforces spatial separation of HOMO and LUMO, promoting rapid IRCT and a competitive oxidation pathway: co-reactant-mediated oxidation at low overpotential followed by direct COF oxidation at higher potential. CN-COF_Py-Bpy_, in contrast, operates solely through the former route. These findings establish atomic control of linkage polarity as a design principle for programming charge-flow directionality and multi-modal ECL outputs in D-A COFs.

Based on the orientation of imine linkages, Mao et al. [33] further performed the post-synthetic cyclisation of imine bonds to a fused quinoline ring to lock the linkage backbone (Figure 10c), extending π-delocalization and suppressing vibrational decay, thereby switching on strong emission. The quinoline-fused isomer bearing the “reversed” imine direction (TFPB-BD(OMe)_2_-H) possesses a narrower band gap (higher HOMO) and a markedly larger electrostatic-potential polarity (ESP extremes: −61 and +28 kcal mol^−1^) than its counterpart (TAPB-BD(OMe)_2_-H). This internal electric field drives spatial separation of holes and electrons, accelerates carrier migration along the ordered π-columns, and yields substantially brighter ECL. Thus, band-gap tuning coupled with directional polarity created by linkage isomerism provides a general design lever for maximizing COF-based ECL.

Additionally, Liu et al. [52] established a design principle based on a cis-trans coordination-configuration control metal COF (MCOF) isomerism in thermodynamic and kinetic aspects for amplifying framework ECL while imparting aqueous dispersibility (Figure 10d). Employing 2,4,6-trihydroxybenzene-1,3,5-tricarbaldehyde (Tp) and cis/trans-bis(glycinato)copper(II) (Cu(Gly)_2_) as ligands, Z-MCOF (*trans*) and E-MCOF (*cis*) were isolated via solvent-regulated pathways. Under thermodynamic conditions, trace water in the organic phase templates regular β-ketoenamine connectivity and locks Cu^2+^ into the trans configuration, affording highly crystalline Z-MCOF that self-disperses in water through weakened inter-layer π–π stacking and axial Cu–OH_2_ solvation. Kinetic polymerization at low temperature favors *cis*-Cu(Gly)_2_ nodes whose steric clash with the compact Tp linker blocks *cis* → *trans* isomerization, trapping the metastable E-MCOF. Density-of-states analysis reveals that the trans isomer positions the Cu d-band center closer to the Fermi level, enhancing adsorption and redox catalysis toward S_2_O_8_^2−^ decomposition. Consequently, Z-MCOF delivers a cathodic ECL quantum efficiency of 98.7% (versus [Ru(bpy)_3_]^2+^/S_2_O_8_^2−^) under equimolar Cu, twice that of E-MCOF and the highest yet reported for an MCOF-based cathodic emitter.

#### 2.3.4. Substituted Groups

The inherent designability and post-synthetic modifiability of covalent organic frameworks provide a versatile handle for tailoring high-performance electrochemiluminescence. Recent studies have systematically probed the impact of halogenation and the incorporation of electron-donating or -withdrawing substituents on the ECL output of COFs, demonstrating that precise functional-group engineering can be leveraged to modulate intramolecular charge transfer and emission efficiency. Hou et al. [53] proposed a covalent-halogen predesign strategy that directly embeds heavy-atom p-orbitals into the π-backbone of pyrene-based COFs, thereby mitigating the charge-transport limitations imposed by the strong dipole of imine linkages. Bromination (or chlorination/iodination) strengthens p–π* conjugation with the imine-linked skeleton, lowering the LUMO and elongating the excited-state lifetime by populating C–C antibonding orbitals that facilitate ultrafast charge separation. Relative to their non-halogenated counterparts, the brominated COFs exhibit a 49-fold amplification in ECL intensity under identical conditions, establishing halogen-mediated p–π conjugation as a general, lattice-level tool for accelerating intrareticular charge transfer and expanding the scope of crystalline reticular nanoemitters in high-performance electrochemiluminescence applications. Chu et al. [54] reported that a deliberate outer-sphere charge-transfer strategy is implemented by anchoring electronically graded substituents onto an isostructural COF backbone. Three frameworks (DVA-COF, PDA-COF, and DMTP-COF) are assembled from TAPB and aldehydes bearing vinyl, unsubstituted phenyl, or methoxy groups, respectively. As the electron-withdrawing strength of the pendant functionality increases (OMe < H < vinyl), the frontier orbital manifold is systematically lowered, narrowing the band gap and prolonging fluorescence lifetime. Consequently, the vinyl-decorated DVA-COF exhibits a four-fold longer exciton lifetime and delivers an ECL quantum efficiency of 4.52%, 4.76- and 39.4-fold higher than PDA-COF and DMTP-COF, demonstrating that fine-tuning the electronic microenvironment of the lattice constitutes a powerful lever for amplifying ECL.

#### 2.3.5. Dimensional Regulation

**Figure 11:**
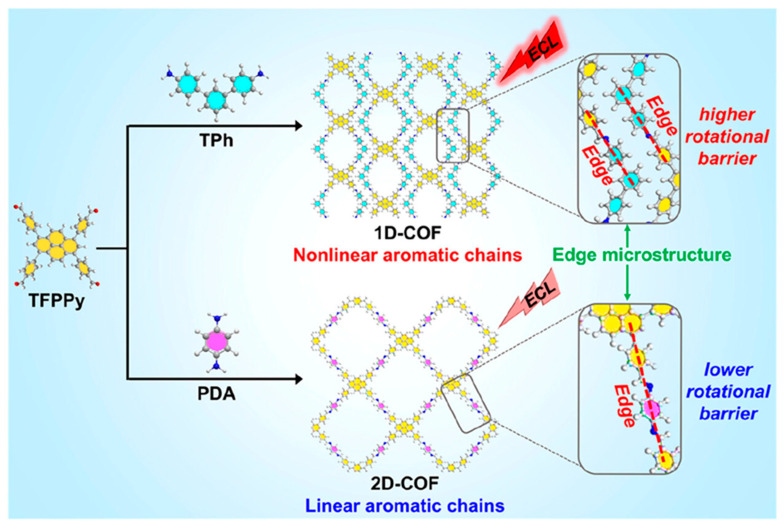
Dimensional regulation for enhanced emission of COF ECL emitter. Reproduced with permission from [55]. Copyright 2024 American Chemical Society.

Dimensional topology of COFs emerges as a critical determinant of ECL intensity, with lower-dimensional architectures exhibiting significantly enhanced emission characteristics compared to their higher-dimensional counterparts. Song et al. [55] unveil a dimensionality-controlled charge-transfer strategy that amplifies ECL by collapsing a pyrene-based lattice into a one-dimensional (1D) dual-chain architecture (Figure 11). The edge-fused ribbons rigidify the aromatic backbone, extend π-conjugation, and minimize energy dissipation, while the constrained torsion suppresses non-radiative decay. Relative to its 2D congener, the 1D-COF delivers 92.5- and 3.2-fold enhancements in anodic (TPrA) and cathodic (K_2_S_2_O_8_) ECL, respectively, with corresponding quantum efficiencies elevated by 2.08- and 3.08-fold. Thus, dimensional down-conversion furnishes a general route to high-efficiency reticular emitters by simultaneously optimizing electronic coupling and conformational rigidity.

### 2.4. Post-Synthesis and Functionalization

COFs possess abundant active sites and readily tunable surface functional groups, enabling precise functionalization with coordinated ions, guest molecules, nanomaterials, and conductive linkers to modulate ECL efficiency and stability. Metallization of COFs is a primary method to integrate metal-based ECL emitters into COF scaffolds for boosting enhanced ECL output. Chiral Ru-complexes, and a series of metal ions (such as Pt^2+^, Pd^2+^, Eu^3+^, Tb^3+^, etc.) can be immobilized onto COF pores by using bipyridine motifs and triazinyl heterocycles, furnishing stable, metal-anchored COFs with amplified and durable ECL emission as well as enabling enantioselective analysis or microreactors [37,38,56,57].

Covalently grafting the co-reactant inside the COF backbone rather than supplying it externally represents a critical way to compress the D-A distance to molecular dimensions and to produce a co-reactant-confined nano-emitter with intense ECL enhancement. For instance, Meng et al. [58] proposed the post-synthetic amidation tethers N,N’-diethylethylenediamine (DEDA^+^) to the C-COF to create a bimodal ECL response ascribed to the first oxidation of covalently bound DEDA^+^ at low-potential and second direct oxidation of the COF skeleton itself at high-potential. The potential and amplitude can be ratiometrically tuned by pH or co-reactant loading, offering a general strategy for amplifying and multiplexing framework-based ECL.

A host-guest strategy is exploited to activate and gate COF-based ECL. The electron-rich host TP-TBDA (TBDA = 2,5-di(thiophen-2-yl)benzene-1,4-diamine) presents pre-fabricated channels that accommodate the electron-withdrawing guest tetracyanoquinodimethane (TCNQ) [59]. Periodic D-A columns are thereby created in a single-crystal-to-single-crystal fashion, establishing an intermolecular charge-transfer network that switches the formerly silent framework into an intense emitter (*Φ*_ECL_ = 79.28% versus [Ru(bpy)_3_]^2+^). The same open channels act as selective recognition sites for UO_2_^2+^ binding, which disrupts the donor ability of TBDA, breaks the charge-transfer pathway, and quantitatively quenches ECL. Thus, guest insertion furnishes a simple yet powerful route to “turn-on” reticular ECL and simultaneously enables analyte-responsive signal modulation.

### 2.5. COF-Based Composites for ECL

COF-based composites constitute a versatile platform for engineering high-performance ECL emitters, either by directly imparting emissive functionality to the framework or by serving as robust scaffolds that amplify and stabilize the emission of guest luminophores. Integrating nanosized metals into COFs can boost the ECL emission to some extent. For instance, AuNCs@COFs exhibit markedly enhanced ECL efficiency, attributed to ligand-suppressed non-radiative decay and diminished self-quenching [60]. Meanwhile, the COF matrix isolates AuNCs within its pores, exposing abundant catalytic sites and accelerating electron transfer to boost peroxidase-like activity. Dual-excitation/emission amplification in AuNCs-COF hybrids was also reported, arising from ligand-locked, level-aligned energy flow [61]. PTCA-COF suppresses non-radiative S_1_ → S_0_ decay of surface ligands by constraining rotational/vibrational modes, while its VB aligns with the AuNC HOMO, injecting hot holes under anodic bias. This synergistically: (i) boosts Au^0^ → Au^+^ oxidation, increasing the population of emissive Au^+^-ligand exciplexes, and (ii) accelerates electrode-to-AuNC electron transfer, elevating excitation rates without accelerating TEA oxidation. Consequently, radiative recombination is intensified solely via excitation enhancement, establishing a general ligand-rigidification/level-alignment paradigm for metal-cluster ECL amplification. Furthermore, a multidimensional amplification strategy was also reported to transform a Tb^3+^-functionalized AIE-COF (Tb@A-COF) into an ultra-efficient ECL nanoplatform. ETBC ligands act as molecular antennae, harvesting excitation energy and transferring it to Tb^3+^ via ligand-to-metal energy transfer, boosting emission by 14.6-fold. A silver-nanowire Schottky heterojunction mechanically buttresses the framework, suppresses π–π collapse and accelerates interfacial charge extraction, doubling signal stability. Cobalt-oxyhydroxide nanosheets provide reversible, concentration-dependent quenching, enabling real-time ECL modulation [62].

Heterostacking COFs with complementary 2D crystals creates van der Waals interfaces that accelerate inter-layer charge transport and confine excitons, delivering synergistic amplification of ECL. For example, a bifunctional MOF/COF heterointerface integrating PTCA-COF (520 nm) and Cu_3_(HHTP)_2_ (600 nm, HHTP = 2,3,6,7,10,11-hexahydroxytriphenylene) delivers strong, non-interfering dual-color ECL [63]. The conductive Cu_3_(HHTP)_2_ phase accelerates electron transfer and lowers activation barriers, simultaneously amplifying both emission channels and catalyzing target recognition. This synergistic dual-enhancement strategy yields a sensitive, ratiometric ECL sensor with robust analytical performance. Han et al. [64] demonstrated a nanoconfinement reactor by assembling luminol-derived carbon dots (Lu-CDs) onto a WS_2_@COF heterojunction that amplifies local radical density at the electrode–electrolyte boundary, delivering unprecedented ECL enhancement. The COF pores impose a domain-limited environment to concentrate H_2_O_2_ at the catalytic edge sites of WS_2_, while the built-in electric field across the WS_2_/COF interface accelerates electron transfer. This synergistic enrichment and catalysis boost both anodic and cathodic ECL intensity and stability beyond that of the free Lu-CDs/H_2_O_2_ system. The platform constitutes an example of co-reactant enrichment-driven radical amplification via nanoconfinement catalysis, offering a general strategy for next-generation, high-sensitivity ECL assays.

### 2.6. Other ECL Enhancement Strategies

Beyond the structural engineering paradigms delineated above, a spectrum of ancillary tactics has been exploited to amplify COF-based ECL. For instance, pre-reduction or oxidation electrolysis of COF-based ECL systems can accumulate radical precursors and effectively stabilize the radical intermediates for efficient ECL processes [35]. Furthermore, judicious selection of reaction solution/electrolyte couples is important for ECL enhancement. Most reported COF-based emitters demonstrate superior ECL emission in aqueous solutions than in organic solvents (acetonitrile, dimethylformamide, tetrahydrofuran, etc.), indicating the crucial role of water in the ECL process [17]. On the other hand, the pH also contributes to the increase in ECL intensity [41]. The deployment of co-reactant accelerators (such as Bu_4_NPF_6_, Ag^+^) is explored to lower the activation barrier for co-reactant oxidation [44,65]. Gold–rhodium core–shell nanoparticles (Au@Rh) were also reported to act as co-reactant accelerators to boost TEA radical formation, synergistically contributing to the spatially resolved ECL imaging [66]. In addition, plasmonic nanoarrays (such as CuS@Ag square-cavity array) have been interfaced with COF ECL probes, wherein surface plasmonic coupling interaction at the metal–organic interface enhances local electromagnetic fields and thereby boosts exciton formation, offering an extrinsic route to ECL signal amplification that complements intrinsic framework optimization [67].

## 3. Applications in Biosensing and Monitoring

COF-based ECL systems represent a transformative analytical paradigm that integrates designable porosity, intrinsic electroactivity, and scalable molecular recognition. Benefiting the brillight ECL performance, COF-based emitters have been explored in biosensing, food safety assay, environmental monitoring and Enantioselective sensing. In this section, the general design and sensing strategies based on COF ECL emitters are discussed with the supporting examples.

### 3.1. Biosensing

COF-based ECL systems represent a transformative analytical paradigm that integrates designer porosity, intrinsic electroactivity, and scalable molecular recognition with ultralow limits of detection (LODs) across diverse biomolecular targets. As shown in Table 1, two design paradigms have been developed for ECL biosensing assay: intrinsically ECL-active COFs that unify signal generation and transduction, COF-immobilized luminophores exploiting nanoconfinement to boost quantum. Combining the tunable ECL on/off modes, biological recognition elements, and signal amplification strategies, COF-based ECL systems demonstrate a broad application in the ultrasensitive assay of nucleic-acid disease signatures, tumor cells or their glycoprotein markers, psychiatric small-molecule drugs, neurochemical metabolites and signaling molecules, and protein biomarkers.

#### 3.1.1. Nucleic-Acid Disease Signatures

**Figure 12:**
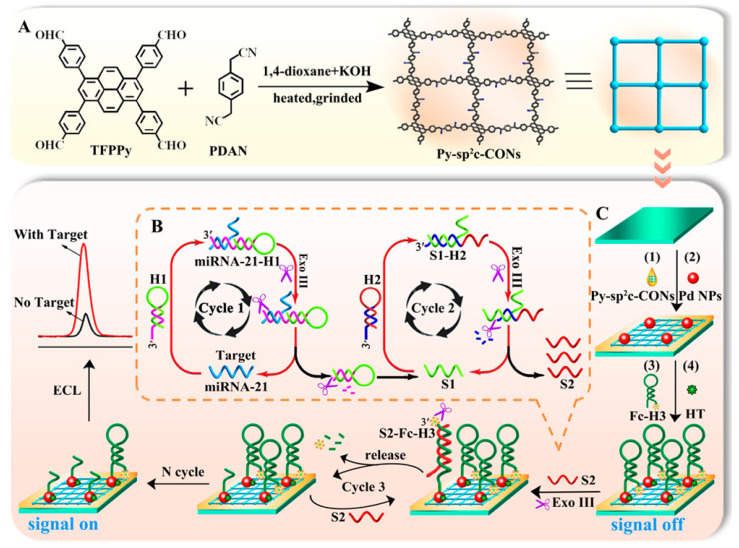
(**A**) Synthesis and structure of Py-sp^2^c-CON. (**B**,**C**) Fabrication of Py-sp^2^c-CON-based ECL system for ECL signal off-on detection of miRNA-21 through enzyme-mediated recycling and target-triggered DNA nanomachinery for signal amplification. Reproduced with permission from [44]. Copyright 2021 American Chemical Society.

The ultrasensitive detection of microRNAs (miRNAs) is of paramount importance in modern molecular diagnostics, as these short non-coding RNAs serve as robust biomarkers for early cancer detection, disease prognosis, and therapeutic monitoring. Coupled with enzyme-mediated recycling and autonomous DNA nanomachinery (walkers, hybridization chain reaction (HCR), catalytic hairpin assembly) for signal amplification, COF-based ECL luminophores have been developed for nucleic acid detection. A novel COF-based ECL sensing platform for specific attomolar-level detection of miRNA-21 was first developed using highly stable Py-sp^2^c-CON as ECL signal reporters with high quantum efficiency and abundant surface-active sites, combined with target-triggered HCR for signal amplification (Figure 12) [44]. The proposed biosensor demonstrates successful application in diluted human serum samples, highlighting its potential as a versatile platform for ultrasensitive detection of low-abundance nucleic acid biomarkers in early disease diagnosis. Furthermore, a TABE-PZ-CON with palladium nanoparticles (Pd NPs) furnishing intense triethylamine (TEA)-coupled ECL was reported for miRNA-21 detection via a robust on-off-on signal alternation [46]. A similar signal on-off-on ECL biosensor was fabricated based on a Ru-MCOF delivering intense baseline ECL coated on glassy carbon electrode (GCE) for ultrasensitive detection of miRNA-155 [40]. Additionally, a plasmonic CuS@Ag square-cavity heterostructure array was deployed to further boost the robust anodic AIECL of ET-COF-COOH with DEDA via the dense electromagnetic hotspots via localized surface plasmon resonance through multiple corner/edge reflections [67]. By virtue of the fast charge separation and bioconjugation of the ECL-active COF and enhanced emission, the resulting ECL sensor quantifies miRNA-124-3p directly in clinical glioma tissues, offering attomolar sensitivity and distinguishing tumor grade with a single biopsy extract.

COF-supported ECL luminophores are also employed to construct ultrasensitive biosensors for assaying nucleic acid signatures. Chelation of Ru^2+^ into cCTFs followed by 4-mercaptopyridine ligation yields SH-Py-Ru-cCTFs, a rigid luminophore whose intra-annular radical annihilation lowers the thermodynamic barrier and boosts ECL efficiency 2.3-fold versus Ru(bpy)_3_^2+^ [37]. Assembled on streptavidin–magnetic beads and wired to a biotinylated anti-miRNA-182 aptamer, the conjugate furnishes a magneto-controlled “on-off-on” ECL aptasensor on a glassy-carbon disk that completes quantification within 41 min, achieves a LOD of 0.28 fM in whole blood, and discriminates low- versus high-grade glioma tissues without RNA extraction. An Eu^3+^-coordinating lanthanide-porphyrin COF microreactor (Eu@TAPP-COF) is forged via Schiff-base condensation, creating cationic nanochannels that spatially confine S_2_O_8_^2−^ oxidation and shorten luminophore–co-reactant distances, delivering enhanced ECL signals [56]. Coupling Eu@TAPP-COF to a catalytic hairpin assembly amplifier yields a label-free biosensor that quantifies miRNA-21 from 100 aM to 100 pM with a 21 aM LOD, offering a streamlined and ultrasensitive route for tumor-marker profiling. A nanoconfinement-driven ECL reactor is engineered based on WS_2_@COF/Lu-CDs for attomolar miR-126 quantitation through a DNA nano-working machine [64]. The resulting signal-off response intensifies linearly with miR-126 concentration, while cascading Boolean AND–YES gates provide multi-layer encryption that shields the assay from external interference and endows the sensing platform with information-security-grade fidelity. Additionally, a natural supramolecular hydrogel (Gel/ZrCl_4_/SBMA) hosting N-dots@COF nanoprobes is reported for exosomal miRNA-381 quantitation [72]. COF nanoconfinement suppresses N-dot aggregation while pre-concentrating co-reactant, and the transparent, ion-conductive quasi-solid electrolyte boosts ECL 1.97-fold versus liquid systems. The integrated sensor attains sensitive detection of miRNA-381 in gastric cancer ascites exosomes, offering a sustainable, mechanics-tough platform for non-invasive malignancy assessment.

#### 3.1.2. Cellular Cancer Entities

Cellular cancer entities are interrogated by integrating epitope-selective capture of circulating tumor cells or their aberrant glycoprotein signatures with the catalytic and energy-transfer tunability inherent to COF-based ECL luminophores, enabling single-cell enumeration and phenotyping within one analytical step. Through synergistic DNA machinery and framework engineering, an amplified ECL aptasensor was fabricated based one a covalent-triazine framework (CTF) emitter and semiconducting Cu_x_Mn_3–x_(HITP)_2_ MOF coupled with an exonuclease III-propelled DNA walker to ultrasensitively detect carcinoembryonic antigen (CEA) from 1 pg/mL to 50 ng/mL with a 2.9 fg/mL LOD [68]. Similarly, an ECL cytosensor based on Au@COF-LZU1@Ru emitter was fabricated, combining a 3-D multivalent aptamer recognition strategy that traps epithelial–mesenchymal-transition-phenotyped circulating tumor cells (CTCs) with an exonuclease-III DNA-walking amplifier [74]. The LOD is down to two cells, counting 2–10^5^ MCF-7 cells/mL. Validated on clinical blood, the assay converts scarce, heterogeneous CTCs into a quantifiable ECL read-out for early diagnosis and therapy monitoring. Additionally, accurate quantitation of the lung-cancer biomarker CYFRA 21-1 at ultra-trace levels was performed in a confinement-enhanced ECL microreactor fabricated by anchoring Ru(dcbpy)_3_^2+^ within a γ-CD-MOF@COF-LZU1 hierarchically porous hybrid [73]. The hydrophobic cavity of cyclodextrin-MOF to pre-concentrate the co-reactant tripropylamine and the shorten electron-transfer distances due to the cationic COF shell synergistically suppresses luminescence self-quenching, boosting ECL efficiency 4.8-fold versus the isolated complex. Coupling this emitter to a magnetic immunosandwich assay, the sensor detects CYFRA 21-1 from 0.05 pg/mL to 50 ng/mL with a 15 fg/mL LOD and successfully stratifies early-stage non-small-cell lung cancer patients from healthy controls. By translating host-guest confinement into amplified photonic output, the study establishes a generalizable strategy for pushing ECL immunoassays into the single-molecule regime and offers a simple, reagent-light platform for routine cancer-marker monitoring.

#### 3.1.3. Protein Biomarkers

Harnessing an integrated recognition–confinement–transduction cascade, COF-based ECL platforms are rapidly emerging as ultrasensitive tools for protein biomarker detection. A nanoplatform that couples a sp^2^-carbon-linked DMTA-TCPB COF with ultrathin CoOOH nanoflakes was proposed for the ECL and electrochemical dual-mode detection of alkaline phosphatase (ALP) [69]. CoOOH simultaneously quenches the bright ECL emission of DMTA-TCPB COF emitter through resonance energy transfer (RET) and amplifies an o-phenylenediamine oxidation peak in differential-pulse voltammetry. ALP-catalyzed hydrolysis of L-ascorbic acid-2-phosphate generates ascorbic acid that reduces CoOOH to Co^2+^, dismantling the nanoflakes and triggering concomitant ECL “turn-on” and DPV “turn-off”. This self-correcting ratiometric response yields a 0.02 U/L LOD in serum. A hierarchically porous microreactor based on imine-linked Ru–MCOF with extended π-conjugation and enhanced internal/external emitter excitation, which compresses the electron/co-reactant diffusion length and electrochemically activates luminophores, for ECL signal-off detection of cardiac troponin I (cTnI) that is an imperative biomarker for acute myocardial infarction, especially in the early diagnosis [70]. Upon cardiac troponin I (cTnI) binding, a proximity-initiated Mg^2+^-dependent DNAzyme cascade is launched, assembling Fc-labeled duplexes to quench the Ru–MCOF emission. Concurrently, a dual-DNAzyme feedback loop that continuously regenerates cleavage substrates and amplifies the quenching event. The integrated nanoreactor–amplifier hybrid detects cTnI across 0.05–50 pg/mL with a 15 fg/mL LOD, offering a single-step, reagent-light route for ultrasensitive cardiac injury profiling.

COF-supported ECL systems are also developed for protein biomarker detection. An ultrasensitive ECL biosensor was proposed with the integration of aminated luminol derivative (*N*-(4-aminobutyl)-*N*-ethylisoluminol, ABEI) as a highly efficient ECL luminophore into COF-LZU1 for sensitively detecting cytochrome c (cyt c) by leveraging aptamer–cDNA hybridization as a selective, label-free recognition element [75]. The biosensor operates in a “signal-off” ECL mode that quantifies cyt c across 1.00 fg/mL~0.10 ng/mL with a LOD of 0.73 fg/mL in human serum samples. These analytical figures of merit establish a robust blueprint for ultrasensitive clinical assays of disease-relevant protein biomarkers. A pocket-sized, two-AA-battery ECL chip is reported for naked-eye prescreening of Alzheimer’s disease (AD) [76]. A current-balancing bipolar electrode couple integrates a TPB-DVA COF-amplified cathode that captures amyloid-β (Aβ) and a low-voltage (3.0 V) anode loaded with Ir(ppy)_3_/Ru(bpy)_3_^2+^ and TPrA. Faradaic current rise upon Aβ binding shifts the anodic emission from green (0.5 V) through yellow (0.7–0.8 V) to red (1.1 V), yielding an R/G ratio that scales linearly with Aβ over 0–15 pM (LOD 1 pM). The color transition (green → yellow → red) is instantly visible, permitting smartphone-based quantitation and point-of-care triage of AD risk without external instrumentation. A reversible signal “off-on” ECL transducer is reported for acetylcholinesterase (AChE) assay, which exploits the AuNPs-decorated Py-PB-COF (Au@COF) as an energy-transfer acceptor to silence luminol emission at a Fc-modified anode [77]. Electro-oxidation of luminol generates an intense anodic ECL whose RET to Au@COF quenches the optical read-out. Anchoring the AChE-aptamer-tagged Au@COF onto the electrode via DNA hybridization establishes the off-state; target binding displaces the composite, restores luminol luminescence, affording a 0.5 nM–1 µM dynamic range and a 170 fM LOD. The prototype combines COF-engineered energy alignment with aptamer specificity, underscoring the broad utility of COF composites for ultrasensitive ECL bioassays.

#### 3.1.4. Bioactive and Signaling Molecules

Dynamic monitoring of bioactive molecules in metabolic micro-events is now accessible through ratiometric ECL biosensors that couple COF-luminophore heterojunctions for affording their ultrasensitive quantitation. These hybrid interfaces behave simultaneously as 3D co-reactant reservoirs, tunable scaffolds, and molecular recognition lattices, positioning ECL as a high-bandwidth modality for decoding the chemical kinetics of living metabolic micro-events. For instance, Song et al. [55] employed an ECL-active 1D COF with outstanding activity to develop an ultrasensitive dopamine biosensor relying on electro-oxidation coupled with ECL RET for dopamine (DA) recognition. An ECL biosensor is constructed based on the anodic ECL generated from the annihilation of the complementary radical ions (COF˙^+^ + COF˙^−^ → COF*) via the processive anodic oxidation and cathodic reduction of TFPPy-DMeTHz-COF for glucose assay [41]. Furthermore, a “signal-on” ECL architecture is described for determining progesterone (P4) that exploits glutamate-animated COF nanoreactors decorated with Pd nanoparticles (Pd NPs@COFs) to amplify RuP/TPrA emission [78]. Coupled with the exonuclease III-propelled DNA walker amplification, the dual amplification enables the progesterone quantitation down to 0.45 pM with high selectivity and operational stability. An ultrasensitive immunosensor for asthma biomarker thymic stromal lymphopoietin (TSLP) by integrating a dual-luminescent T-COF constructed from AIE-active monomer and 9,10-anthracenedicarboxaldehyde units into a microfluidic chip and pairing it with camel-derived nanobodies [65]. Target binding recruits an Ag^+^-intercalated dsDNA label; Ag^+^ catalyzes radical generation, producing a positive ECL read-out from 1 pg/mL to 4 ng/mL. Harnessing COF rigidity to drive AIECL while exploiting nanobody precision, the platform exemplifies how framework-confined molecular rotors and next-generation affinity reagents can jointly deliver clinical-grade sensitivity for respiratory disease monitoring.

Additionally, the COF-based ECL platform also finds its application in the reliable detection of adeno-associated virus serotype 8 (AAV8), which is the gene therapy vector for in vivo gene delivery with high tissue specificity. Du et al. [66] introduced a nanobody-ECL imager that merges a TPE-cored COF (TC-COF) with aggregation-induced, photostable, high-quantum-yield anode signal and Au@Rh core–shell nanocatalysts that accelerate TEA radical formation for an additional 8.3-fold brightness gain. Camelid nanobodies, oriented via C-terminal cysteine–Au coupling, provide sub-nanomolar affinity and 50% smaller footprint than IgG, ensuring unobstructed epitope access on the AAV8 capsid. Binding events are visualized directly on a pixel array; grey-scale integration furnishes a digital calibration curve spanning 1 × 10^8^–5 × 10^11^ vg/mL with an LOD of 1 × 10^7.15^ vg/mL. This first deployment of AAV8-specific nanobodies in an ECL format offers a reagent-light, single-step protocol for vector biodistribution mapping and therapeutic-dose confirmation.

#### 3.1.5. Psychiatric Small-Molecule Drugs

Developing ultrasensitive, selective, and portable detection platforms for psychiatric small-molecule drugs is a prerequisite for mitigating the ongoing opioid crisis and for real-time surveillance of emerging new psychoactive substances. COF-driven ECL architectures are emerging as the focal point for reagent-light, single-step surveillance of psychiatric small-molecule therapeutics, offering high sensitivity and direct readout in complex fluids. On-site detection of the designer benzodiazepine diclazepam was reasonably proposed based on a pocket-sized ratiometric ECL sensor that uses a single, dual-emissive MOF/COF film: a blue-emitting Zr-MOF matrix threaded with a red-emitting PTCA COF guest [63]. Diclazepam partitions specifically into the PTCA COF nanochannels and quenches the red emission via photo-induced electron transfer while the blue signal remains invariant, yielding an internal reference that is immune to matrix effects. The device quantifies diclazepam in 50 µL undiluted serum from 0.1 nM to 10 µM (LOD 30 pM) and distinguishes the drug from fifteen confounding benzodiazepines. By converting host–guest recognition into a self-calibrated color switch, the study provides a reagent-light, smartphone-readable tool for real-time surveillance of emerging psychoactive substances and illustrates how engineered heteropore frameworks can simultaneously advance selectivity and sensitivity in complex biofluids. Furthermore, a ternary ECL architecture that interfaces a PTCA-COF luminophore with Ag@CuCo_2_O_4_ nanorod co-reaction accelerators to achieve sub-picogram quantitation of the designer cathinone 4-chloroethcathinone (4-CEC) [71]. The spinel oxide couple (Co^3+^/Co^2+^ and Cu^2+^/Cu^+^) catalytically decomposes S_2_O_8_^2−^, while Ag nanoparticles and the nanorod’s intrinsic hydrogen-evolution capability synergistically expedite proton relay, collectively amplifying the “signal-on” ECL baseline. Target binding triggers an Fc-tagged aptamer to fold into a stem-loop, positioning the ferrocene quencher within the nanorod tunnel and instigating a distance-dependent “signal-off” response. This conformation-gated quenching furnishes a 1 pg/L µg/L linear window and a 0.25 pg/L LOD, illustrating how multi-effect catalytic COF hybrids can translate aptamer mechanics into ultrasensitive surveillance of emerging psychoactive substances. Additionally, terbium-centered A-COFs are engineered into an ultrasensitive ECL nanoantenna for screening isobutyryl fentanyl (iBF) by converging three orthogonal amplification strategies [62]. This multidimensional synergy yields a stable, intensity-boosted ECL read-out that resolves iBF down to 0.90 fg/L, establishing rare-earth COFs as next-generation platforms for ultra-trace analyte surveillance.

The convergence of designer porosity, intrinsic electroactivity, and scalable surface engineering positions COF-ECL platforms as next-generation biosensors capable of unifying genomic, proteomic, and metabolic information, thus promising holistic profiling for precision medicine. Despite remarkable sensitivity, challenges remain in standardizing biofunctionalization, achieving long-term stability in raw clinical samples, and developing predictive models linking COF topology to ECL performance—limitations that currently constrain translation from proof-of-concept to routine clinical diagnostics.

### 3.2. Food Safety Assay

Heightened consumer awareness of food safety has transformed ultrasensitive quantification of antibiotic, pesticide, and carcinogenic residues into a critical analytical imperative, while accurate profiling of key nutrients is now equally essential for ensuring nutritional quality. In response, COF-based ECL platforms, either employing the framework itself as the emitter or as a support for immobilizing luminophores, are being intensively investigated for food-safety applications and are demonstrating unprecedented sensitivity, selectivity, and stability across diverse food matrices. Here, molecularly imprinting and aptamer recognition are the main sensing strategies for specific analyte assays in the food safety field, as summarized in Table 2.

AIECL-based COF lumophores are harnessed as signal transducers for ultrasensitive detection of antibiotic residues in complex food matrices. For instance, an ultrasensitive ECL nano-sensor for ciprofloxacin (CFX) is fabricated by integrating the COF-AIECL with nanozymatic Fe_3_O_4_@Pt nanoparticles and a surface-imprinted polymer [79]. COF-AIECL delivers intense ECL that is further amplified by the peroxidase-mimetic Fe_3_O_4_@Pt nanozyme. A molecularly imprinted film (*o*-aminophenol/CFX) is electropolymerized onto the modified GCE; template removal yields selective cavities whose CFX rebinding quenches the ECL signal. Under optimal conditions, the sensor exhibits a 2–3000 pM linear range and a 0.598 pM detection limit while retaining excellent selectivity, stability, and reproducibility. Successful quantification of CFX in complex food matrices validates the platform as a powerful tool for residue monitoring. A similar strategy is also applied in detecting chloramphenicol (CAP) by using CAP-MIP/COF-AI-ECL/Co_3_O_4_/Au as a signal emitter. Additionally, a Zr-coordinated, amide-linked porphyrin 2D COF with graphene-like multilayer architecture, lower band gap (1.6 eV), and atomically ordered π-columns is reported to support luminol for ECL assay [86]. Zr(IV) centers embedded in the porphyrin cores act as catalytic hotspots, endowing the framework with metallic in-plane conductivity and exceptional electrocatalytic activity. When harnessed as the recognition element in a gate-controlled, molecularly imprinted ECL sensor, the Zr-amide-Por COF delivers a linear response to tetracycline from 5 to 60 pM with a 2.3 pM detection limit, offering a practical route to on-site monitoring of food safety.

COF-based ECL platforms are also emerging as powerful tools for the ultrasensitive detection of pesticide residues in complex food matrices. A D-A TP-ML COF served as both an ECL reporter and a RET donor to construct a malathion (Mal) biosensor in which AuNPs act as the quencher [32]. Polyacrylamide gel electrophoresis analysis confirms the single-enzyme-assisted CHA double-cycle amplification mechanism. The resulting platform exhibits excellent specificity and stability, a linear range of 0.001–100 ng/mL, and a detection limit of 19.03 fg/mL, highlighting its potential for ultrasensitive organophosphorus pesticide detection. Song et al. [42] proposed a novel ECL biosensor for Mal detection based on a signal-off strategy. ECL RET from the excited TFPPy-TPh-COF donor to ZIF-8 affords near-complete ECL quenching, while acetylcholinesterase (AChE) hydrolyses acetylcholine to acetic acid, dissolving ZIF-8 and recovering emission. Mal inhibits this cascade, restoring the quenched state. The resultant biosensor exhibits a LOD of 2.44 pg/mL and is readily extendable to other organophosphates (methidathion, chlorpyrifos, paraoxon) with validated quantification of Mal in fresh produce (pak choi, lettuce, apple). A CRISPR/Cas12a-driven ECL biosensor is reported for ultrasensitive detection of acetamiprid by employing PTCA-COF as a robust ECL emitter whose ordered π-columns and nanoconfined pores suppress non-radiative decay and deliver stable cathodic emission [43]. Au-functionalized Fe_3_O_4_ magnetic beads (Au@MBs) are employed as spherical nucleic-acid scaffolds to enrich acetamiprid-aptamer/activator-DNA duplexes. Upon target binding, the released activator unleashes Cas12a trans-cleavage activity, detaching ferrocene-labelled quenchers from the electrode surface and restoring the ECL signal. This “off-on” strategy achieves sub-femtomolar sensitivity and demonstrates a general blueprint for pesticide quantification in complex matrices. A molecularly imprinted ECL sensor is fabricated for monitoring carbaryl by drop-casting DAFB-DCTP@carbon-nitride nanosheets (CNNs) onto a GCE, followed by the electropolymerization of carbaryl and o-phenylenediamine [80]. The D-A COF/CNN heterojunction generates intense cathodic ECL with K_2_S_2_O_8_, but the insulating MIP film blocks electron transfer and quenches emission. Template extraction re-opens the transport channel, restoring ECL intensity and yielding an MIPs/COFs@CNNs/GCE probe capable of sensitive carbaryl quantification in complex matrices.

Rapid, ultrasensitive detection of food-borne toxins and carcinogens is imperative for safeguarding public health, where COF-based ECL sensors are now emerging as the front-line analytical tools to meet this demand. A TFPT-TAPB-COF delivering intense cathodic ECL serves as both capture matrix and signal reporter for detecting zearalenone (ZEN) [81]. ZEN conjugated to BSA (ZEN-BSA) is immobilized via hydrophobic/H-bond/electrostatic interactions, partially blocking electron transfer and establishing the initial “on” state. Upon competitive incubation with free ZEN and Ab/CuFe_2_O_4_@PDA probes, target ZEN displaces the quencher-conjugated antibody; residual Ab/CuFe_2_O_4_@PDA binds to surface ZEN-BSA and imposes trimodal quenching via RET, electron transfer, and radical scavenging, turning the signal off. Increasing ZEN concentration progressively liberates the luminescent surface, restoring emission (“on”). The resulting “on–off–on” ECL immunosensor exhibits a broader dynamic range and a 7.9 fg/mL LOD for ZEN in food extracts, demonstrating the power of integrating D-A COF emitters with multi-mechanism quenchers for ultrasensitive mycotoxin analysis.

Beyond serving as intrinsic emitters, COFs (e.g., COF-LZU-1) functioning as high-surface-area scaffolds that immobilize luminophores and furnish nanoconfined reactors also enable ultrasensitive ECL detection of food-borne toxins. The COF-LZU1 with high surface area and hydrophobic nanochannels is used as a scaffold for immobilizing luminophore Ru(bpy)_3_^2+^ and an ECL micro-reactor for stabilizing TPrA˙ radicals as well as accelerating their electrochemical oxidation. Confinement-enhanced ECL enables an aptasensor for aflatoxin M1 (AFM1) with a six-orders-of-magnitude linear range and 0.03 pg/mL LOD, establishing COF micro-reactors as a general strategy for ultra-bright, stable bioanalysis [21]. Another COF-LZU1-supported COF@Ru ECL platform is also fabricated for highly specific, stable, and sensitive detection of ochratoxin A (OTA) via a CRISPR/Cas12a trans-cleavage gated signal-on ECL pathway [82]. Nanoconfined-structured Ru@COF-LZU1 is also employed for selectively and sensitively detecting a highly carcinogenic contaminant, N-nitrosodimethylamine (NDMA), with aptamer recognition and magnetic separation [83]. The presence of NDMA induces the conformational transform of the DNA-aptamer complex to release Ru@COF-LZU1 in solution, leading to an increased ECL signal with TPrA after magnetic separation. COF-LZU1 further to charge in situ grown CsPbBr_3_ perovskite as nanoluminophore to construct an aptasensor for T-2 toxin cooperating with target-responsive DNA hydrogel containing an anti-T-2 aptamer and alkaline-phosphatase (ALP) cargo [84]. Upon T-2 recognition, the hydrogel collapses and releases ALP to convert AAP into ascorbate (AA) for forming the strongly reducing ascorbyl anion radical (A^−^•, pKa 0.45) at the electrode. The high-energy electron-transfer reaction between simultaneously generated CsPbBr_3_^+^ via oxidation and A^−^• populates the excited state CsPbBr_3_*, whose radiative decay furnishes an amplified ECL signal proportional to T-2 concentration. The platform exhibits a wide dynamic range and sub-pg/mL detection limit, validating COF-confined perovskite ECL for ultrasensitive mycotoxin monitoring. Additionally, a quench-mode molecularly imprinted ECL sensor is proposed by integrating CsPbBr_3_ quantum dots with a COF-300-Au composite scaffold and a cross-linked 3-thiopheneacetic-acid-functionalised AuNP imprinting layer for ultrasensitive detection of the priority pollutant benzo[a]pyrene (BaP) [85].

Accurate identification of food nutrients is essential for both product-quality assessment and healthy-diet promotion. Few studies have exploited COF-based ECL systems for this purpose. Cui et al. [87] developed Ru@SiO_2_-CMIPs core–shell nanoprobes comprising Ru(bpy)_3_^2+^-doped SiO_2_ cores as ECL emitter encapsulated within molecularly imprinted COF shells for monitoring the cyanidin-3-O-glucoside (C3G) that is the most abundant anthocyanin in pigmented fruits and vegetables and a functional food ingredient with antioxidant, anti-inflammatory, and cardioprotective properties. The crystalline COF matrix acts simultaneously as a permeable barrier that prevents Ru(bpy)_3_^2+^ leaching and a nanoconfined reaction chamber that increases collision frequency between Ru(bpy)_3_^2+^ and TPrA, stabilizing the ECL signal and amplifying emission intensity. After C3G templates are removed, the imprinted cavities enable selective recognition; subsequent C3G binding triggers RET from Ru(bpy)_3_^2+^ to the anthocyanin, producing a quantifiable ECL decrease. This RET-gated, imprint-enhanced strategy affords sub-pg/mL LOD and excellent selectivity for C3G in complex food extracts, illustrating the potential of COF-engineered ECL nanoprobes for accurate nutraceutical analysis.

Despite remarkable analytical sensitivity, several interconnected challenges impede practical translation of COF-based ECL sensors for food safety. More specific recognition of target analytes should be developed besides molecularly imprinting and aptamer recognition. Particularly, the spectral congestion of COF emitters coupled with non-specific molecularly imprinting polymer cross-reactivity for structural analogs (e.g., tetracyclines, organophosphates) precludes simultaneous multi-residue screening. Furthermore, mechanistic understanding is incomplete to guide the rational design of COF-based ECL systems. Finally, regulatory approval is stalled by uncharacterized nanomaterial leaching, uncertain toxicological profiles, and deficient inter-laboratory validation, preventing routine adoption.

### 3.3. Environmental Monitoring

ECL sensors are rapidly becoming indispensable tools for field-scale environmental surveillance. Reticular-emitter COF-based ECL platforms provide real-time alerts for priority pollutants such as nuclear species, heavy-metal ions, pesticides, antibiotic residues, etc., in natural waters, effluents, and soils. As shown in Table 3, advanced COF-based ECL sensors have been developed for ultrasensitive detection of radiative and/or nuclear species in the surrounding water by tuning the charge transfer during the chelation reaction or specific recognition unit. Jiang et al. [59] reported a host-guest D-A structure based on TCNQ-decorated TP-TBDA COF serving as an ECL emitter and a selective recognition site for uranyl ions (UO_2_^2+^) binding and detection. The bound UO_2_^2+^ disrupts the donor ability of TBDA, breaking the charge-transfer pathway and quantitatively quenching ECL. Cui et al. [47,49] converted the crystalline BTT-TBTN and BTT-DCTP COFs to their amidoxime analogues (BTT-TBTN-AO and BTT-DCTP-AO) by treatment with excess NH_2_OH·HCl and triethylamine for ECL detection of UO_2_^2+^. By virtue of two strategies: molecularly imprinting recognition and chelation-induced charge transfer, highly specific and sensitive monitoring of UO_2_^2+^ is achieved with an ultra-low LOD that is two orders of magnitude below the best reported luminescent UO_2_^2+^ probe and surpasses all previously reported ssDNA-modified ECL platforms, underscoring its exceptional sensitivity. Luo et al. [50] reported that systematic augmentation of framework nitrogen progressively lowers both reduction potential and emission energy of TBTN-N*_x_*-COFs (*x* = 0–3). A sensitive ECL sensor was developed for lutetium ion (Lu^3+^) detection in aqueous solutions based on the quantitative change in ECL signal deriving from the strong affinity of triazine core in TBTN-N_3_-COFs to Lu^3+^. Additionally, Chu et al. [54] proposed a signal-off strategy for detecting the iodine ions and iodixanol based on the competing charge transfer of iodine redox species with the TPrA radicals against DVA-COF radicals for forming a steady excited state.

COF-based ECL platforms exploit framework-embedded chelators or recognition motifs to sequester heavy-metal ions. Competitive electron transfer upon ion binding produces a quantitative quenching response, enabling femtomolar screening without external labels. By completing the electron transfer reaction, heavy metal ions can quantitatively quench the ECL emission by binding to the COFs. Following this strategy, Hu et al. [88] proposed a Ru(dcbpy)_3_^2+^-functionalized COFs (RuCOFs) by stitching 4,4′,4″-(1,3,5-triazine-2,4,6-triyl)triphenylamine with 2,2′-bipyridine-5,5′-diamine, yielding a high-surface-area lattice rich in N,N′-bipyridine chelates. These sites act as dual-function recognition motifs: Hg^2+^ binding positions its HOMO within the RuCOF band gap, enabling excited-state electron transfer and luminescence quenching (LOD = 4.71 nM), whereas Zn^2+^ coordination nucleates 3D nanoflowers that enlarge the active area and catalyse SO_4_˙^−^ generation, producing a signal enhancement (LOD = 6.57 nM). The opposing responses across 1 µM–1 nM afford a ratiometric, single-platform sensor for simultaneous Hg^2+^/Zn^2+^ detection. Similarly, Mao et al. [33] also performed the ultrasensitive detection of As(V) ions in water by using the quinoline-fused TFPB-BD(OMe)_2_-H with enhanced ECL emission. On the other hand, specific aptamer recognition is also employed for developing high-performance and selective detection of targeting heavy-metal ions (e.g., Pb^2+^, Cd^2+^) in soil or water [60,61].

**Figure 13:**
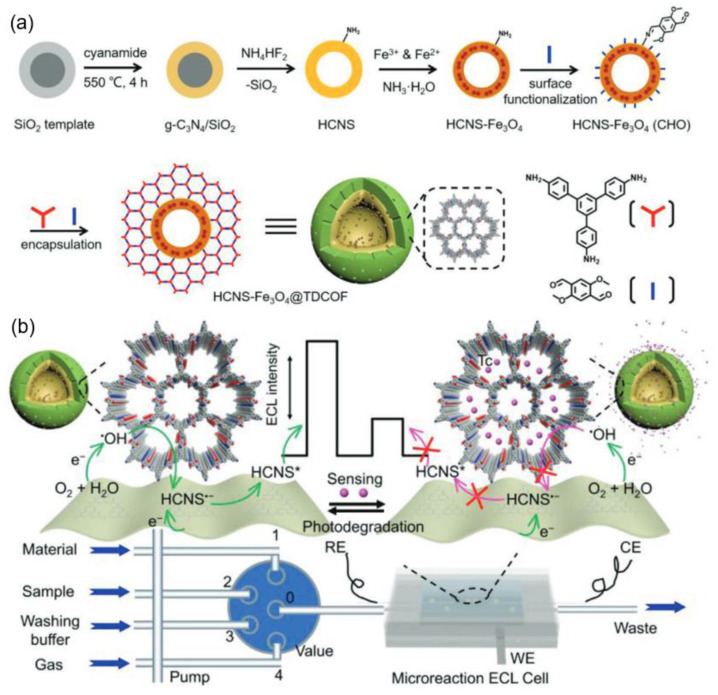
Schematic diagram of (**a**) the synthetic procedure for HCNS-Fe_3_O_4_@TDCOF and (**b**) its ECL platform for monitoring the photodegradation of Tc. Reproduced with permission from [89]. Copyright 2022 Wiley-VCH.

COF-based ECL architectures also demonstrate record sensitivities and lower LODs toward a broad spectrum of organic micropollutants (e.g., antibiotics, antineoplastics), positioning them as next-generation field-deployable platforms for real-time environmental surveillance. A biomimetic, self-responsive ECL platform is reported for in situ monitoring of photodegradation kinetics [89]. A crystalline, porous heteroshell of imine-linked COFs grown epitaxially on Fe_3_O_4_-decorated polymeric hollow g-C_3_N_4_ nanospheres (HCNS–Fe_3_O_4_@TDCOF) functions simultaneously as a visible-light photocatalyst and an O_2_-co-reactant ECL indicator, as show in Figure 13. Tetracycline (Tc) quenches the HCNS/oxygen ECL signal: the resultant intensity decreases linearly with log *C*_Tc_ (0.10–10 µg L^−1^, R^2^ = 0.9943) and affords a sub-ppb detection limit of 0.031 µg L^−1^. This performance originates from the nanoconfined cavity of TDCOF, which concentrates Tc and dissolved O_2_ at the catalytic interface, amplifying both photocatalytic degradation and ECL transduction in a single self-reporting nanoreactor. An aptamer recognition strategy is also proposed to screen the antibiotic oxytetracycline (OTC) in lake water with a lower LOD by employing the ZnCdS@TAPB-DMTP-COF/GCE platform [90]. Cao et al. [51] developed a sensitive ECL sensor for detecting antineoplastic doxorubicin (DOX) based on the ECL resonance energy transfer between DOX and excited NC-COF_Py-Bpy_. Additionally, Ma et al. [91] constructed Pt-nanoparticle/CD-COF on the GCE surface to generate a robust ECL read-out for detecting bisphenol A (BPA). Anchoring Fc-labelled ssDNA (Fc-SH-DNA) via Pt–S bonding quenches the emission (“off”). Target BPA activates the CRISPR/Cas12a trans-cleavage machinery, releasing Fc from the interface and restoring cathodic ECL (“on”). This transduction cascade converts femtomolar BPA recognition into a quantifiable light signal, affording an ultrasensitive “off–on” biosensor with sub-ppt detection capability.

COF-based ECL systems for environmental monitoring remain in their infancy, characterized by limited case studies and underdeveloped detection capabilities for hazardous ions and molecules. Critically, the fundamental mechanisms governing analyte recognition and signal transduction are not yet fully elucidated. complex matrices (natural waters, effluents) introduce competing ions, organic matter, and particulates that compromise selectivity, while structural analogues of target pollutants cause unresolved cross-reactivity, precluding multi-residue screening. To advance beyond proof-of-concept, systematic investigation must focus on enhancing material stability and functional versatility under variable field conditions, while concurrently expanding the scope of applicability through rigorous validation across diverse environmental matrices. This necessitates comprehensive studies on long-term operational stability, resistance to biofouling and chemical degradation, and the development of robust, regenerable sensor architectures capable of sustained performance in complex, real-world effluents.

### 3.4. Enantioselective Sensing

**Figure 14:**
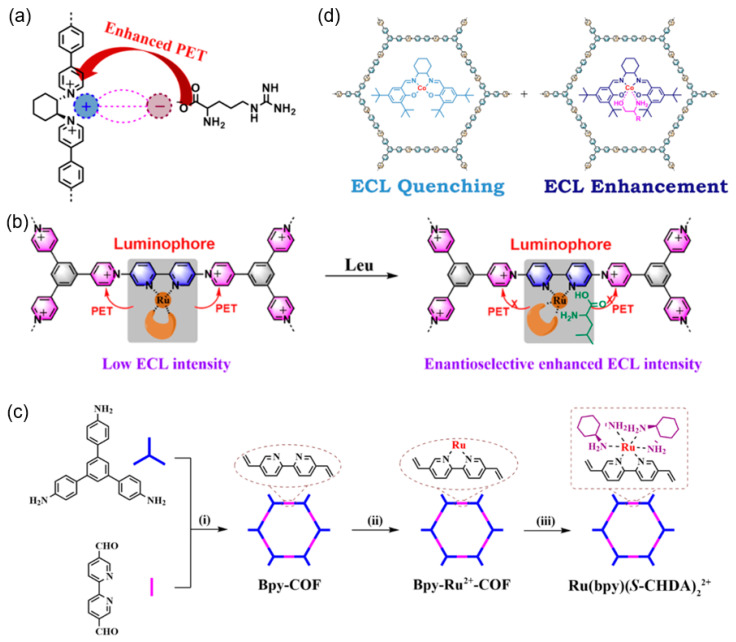
(**a**) ECL quenching mechanism based the enhanced PET between triPhPy^+^-(*S*)-CHA and D-Arg. Reproduced with permission from [93]. Copyright 2024 American Chemical Society. (**b**) The possible chiral recognition mechanism for (*R*)-Ru-L-Leu. Reproduced with permission from [57]. Copyright 2024 American Chemical Society. (**c**) The synthesis of Ru(bpy)(*S*-CHDA)_2_^2+^ for enantioselective sensing. Reproduced with permission from [94]. Copyright 2024 American Chemical Society. (**d**) Differentiated ECL emission based on iCOF-hosted Co(III) complex. Reproduced with permission from [95]. Copyright 2024 American Chemical Society.

The integration of stereoselective recognition sites into COF backbones converts enantiomeric structural differences into quantifiably distinct ECL signals, affording a single-output platform for high-fidelity chiral discrimination that advances the practical utility of COF-based ECL sensors. Covalent installation of chiral building blocks into crystalline COF lattices directly harnesses their ordered π-backbones and intrinsic ECL to convert enantiomeric recognition events into differential optical signals, providing a concise and robust route to enantioselective assay, as summarized in Table 4. Ruan et al. [92] reported a chiral 1D ionic COF synthesized via co-condensation of π-conjugated PTCDA with (*R*)-2-methylpiperazine. This framework exhibits a bidirectional ECL response to enantiomers, enhancing the signal for D-penicillamine and quenching it for L-penicillamine. Theoretical simulations reveal that the narrower HOMO–LUMO gap in COF-D-PA facilitates electron transition and enhances ECL emission, whereas the larger gap in COF-L-PA results in less efficient excitation and a weaker signal. This sensor enables both identification and quantification of chiral molecules based on ECL signal modulation, offering a novel method for detecting and quantifying chiral compounds in pharma, diagnostics, and environmental monitoring. Similar strategy is also applied by Yuan et al. [93] for constructing an ECL-active ionic COF (iCCOF) triPhPy^+^-(*S*)-CHA, which exhibits enantioselective ECL quenching towards amino acid enantiomers, including arginine (Arg), alanine (Ala), leucine (Leu), and lysine (Lys), due to enhanced photoinduced electron transfer (PET) from the formed complex via electrostatic attraction. At pH 6.5, triPhPy^+^-(*S*)-CHA shows a 33.0-fold higher ECL intensity for D-Arg (15.7k au) compared to L-Arg (446 au) at −1.79 V, attributed to the diastereomeric adducts formed between the chiral [Py]^+^ and Arg’s -COO-, which amplifies PET and quenches ECL (Figure 14a). Below pH 5.5, recognition efficiency drops due to the instability of the radical cation in acidic conditions, preventing effective interaction between Arg’s -COOH and [Py]^+^. Conversely, the inverse-configured iCCOF triPhPy^+^-(*R*)-CHA demonstrates opposite enantioselectivity for Arg enantiomers under the same conditions.

Covalent grafting, metal coordination, and host-guest chemistry have all been deployed to implant enantiomer-discriminating motifs within COF scaffolds, furnishing chiral interfaces that translate stereoselective recognition into quantifiable ECL signals for robust enantioselective sensing. Song et al. [36] established a β-cyclodextrin (β-CD) modified aminal-linked COF for discriminating L-phenylalanine (L-Phe) against D-Phe via the competitive host-guest interaction between β-cyclodextrin (β-CD) and different guest molecules (Rhodamine B (RhB) and Phe enantiomers) for modulating ECL emission. RhB quenches the ECL of the aminal-linked COF via resonance energy transfer. L-Phe can displace RhB from the β-CD cavity with higher binding affinity than D-Phe, restoring bright ECL. Conversely, D-Phe fails to displace RhB, maintaining lower ECL emission. Tan et al. [57] reported a chiral ionic COF platform with (*R*)-configured Ru-ligand coordination for enantioselective ECL sensing (Figure 14b). With the rapid electron transfer of the ionic COF scaffold, the chiral ECL emitter exhibits distinct ECL responses to L- and D-enantiomers in the presence of Na_2_S_2_O_8_, where D-amino alcohols and L-amino acids yield higher signals, with ECL intensity ratios (L/D) ranging from 1.25 to 1.94. This uniform recognition pattern correlates with concentration and pH, reflecting structural changes in the analytes. DFT calculations reveal that (*R*)-Ru-L-Leu is more stable than (*R*)-Ru-D-Leu by 1.4 kJ mol^−1^, underpinning the observed enantioselectivity. The ECL mechanism involves suppression of PET upon complexation with amino acids/alcohols, which enhances steric hindrance around the Ru-ligand. This work establishes a general strategy for designing chiral COFs that translate molecular chirality into differential ECL signals for efficient enantioselective analysis. Yuan et al. [94] report a chiral ECL sensor based on Ru(II) coordinated dipyridyl-functionalized COFs and enantiopure cyclohexane-1,2-diamine (Figure 14c). This ECL-active unit exhibits strong emission in the presence of K_2_S_2_O_8_. When immobilized on a glassy carbon electrode, it differentiates between L- and D-enantiomers of five amino acids (Trp, Leu, Met, Thr, His) via competitive interactions. At pH 5.5, L-Trp shows 1.57-fold higher ECL intensity than D-Trp, attributed to electrostatic interactions between (*S*)-CHDA and Trp’s -COOH group. Conversely, (*R*)-CHDA coordination yields higher ECL for D-Trp. Nonpolar Leu and Met favor L-enantiomer ECL, while polar Thr and His exhibit the opposite trend. The sensor displays good stability and a linear relationship between ECL intensity and the logarithm of concentration, offering a versatile platform for chiral analysis in diverse fields. Additionally, an achiral ionic COF with pyridinium units and clear ECL was used to immobilize a chiral Co(III) complex for enantioselective sensing [95]. The resulting ECL sensor shows distinct responses to amino alcohol enantiomers: (*S*)-enantiomers enhance ECL, while (*R*)-enantiomers quench it, with the highest ECL intensity ratio (47.7) observed for (*S*)- vs. (*R*)-amino alcohols (Figure 14d). The electroactive species TriPhPy^+^-BiPh generates a radical cation upon reduction, which reacts with SO_4_^•−^ to produce ECL, demonstrating the sensor’s high enantioselectivity.

## 4. Conclusions and Perspective

The rational design of COFs for reticular ECL represents a rapidly evolving frontier in electroanalytical chemistry. By integrating structural predictability, tunable π-conjugation, and intrinsic porosity, COFs offer a unique platform to overcome the limitations of traditional molecular ECL emitters. As highlighted throughout this review, the ability to regulate linkage and monomer chemistry, engineer donor–acceptor motifs, control interlayer π–π stacking, embed AIE active sites within a crystalline lattice, and perform post-synthetic modification as well as composite formation have enabled unprecedented gains in ECL efficiency, stability, and signal-to-noise ratio. These advances have translated into ultrasensitive biosensing platforms capable of detecting analytes at the attomolar level, with improved reproducibility and operational robustness.

Despite these achievements, several challenges remain in the design and synthesis aspects. First, while IRCT and AIE mechanisms have been successfully exploited to activate ECL from otherwise inactive building blocks, the structure–property relationships governing excited-state dynamics in COFs remain poorly understood. The ECL performance of the pristine COFs is still lower than that of post-synthetics or composites due to the poor intrinsic conductivity. Constructing fully π-conjugated planar frameworks or introducing conductive linkages is helpful to enhance their charge transport and ECL efficiency. More sophisticated theoretical models and in situ spectroelectrochemical studies are needed to unravel the interplay between electronic band structure, exciton localization, and co-reactant diffusion within the framework. Second, achieving precise control over crystallinity, porosity, and hierarchical structures (e.g., hollow nanostructures, vesicles) is challenging but crucial for optimizing ECL properties. So far, most COFs for ECL systems still rely on solvothermal synthesis, which limits scalability and integration into miniaturized devices. Green, ambient-condition synthetic protocols, such as interfacial polymerization, vapor-assisted crystallization, or mechanochemical approaches, are urgently needed to facilitate real-world deployment.

Although COFs exhibit excellent thermal and chemical stability, their long-term performance in complex biological matrices remains underexplored. Future work should prioritize biocompatibility assessments, antifouling surface engineering, and the development of wearable or implantable ECL devices. On the other hand, their application is still in the early stages, while COF-ECL systems have shown promise in environmental, food, and biomedical sensing. Additional strategies are still eager for constructing novel biosensing recognition elements, and further work is needed to expand their versatility and real-world utility. Inspired by the CRISPR-based recognition, DNA nanomachines, and smartphone-based readout systems in recent works, the integration of COFs with emerging technologies could unlock new paradigms in point-of-care diagnostics and environmental monitoring.

Looking forward, the convergence of reticular chemistry with ECL science holds transformative potential. By integrating machine learning (ML)-guided monomer library screening with advanced synthetic and characterization pipelines, next-generation COF nanoemitters featuring programmable emission profiles, multi-analyte discrimination, and self-calibrating ratiometric outputs are now within reach [96,97,98,99]. Central to this evolution is a paradigm shift from single-property prediction toward multi-objective optimization frameworks that concurrently resolve intrinsic trade-offs among luminescence quantum yield, charge-transfer kinetics, and electrochemical durability. Inverse design via generative models (VAEs/GANs) must retrospectively map target ECL performance metrics—spanning excited-state energetics and co-reactant synergy—onto optimal building blocks and stacking sequences, circumventing prohibitive TD-DFT costs through transferable fragment-based descriptors. Complementarily, active learning loops coupling automated electrochemical synthesis with in situ ECL feedback will enable few-shot model refinement, while AutoML platforms democratize screening by automating descriptor selection and architecture optimization for data-scarce regimes. Crucially, explainable AI must decode non-intuitive structure–property mechanisms to guide rational defect engineering, though these advances remain fundamentally contingent upon systematic database curation that synergizes high-throughput computational screening with rigorous experimental validation. Ultimately, the continued evolution of COF-based ECL systems will not only redefine the limits of analytical sensitivity but also expand the scope of electrochemiluminescence into new realms of personalized medicine, food safety, and environmental sustainability.

## Figures and Tables

**Table 1:** Summary on the COF-based ECL lumiphores and COF-supported ECL systems for biosensing.

App.	COF	ECL System	Co-Reactant	Sensing Mechanism	Analytes	Linear Range	LOD	Samples	Ref.
COF-based ECL lumiphores	Py-sp^2^ C-CON	Py-sp^2^ C-CON/S_2_O_8_^2−^/Bu_4_NPF_6_	K_2_S_2_O_8_ with Bu_4_NPF_6_ as accelerator	DNA/RNA recognition	microRNA-21	100 aM~1 nM	46 aM	extracts of cancer cells (HeLa, MCF-7)	[44]
	TABE-PZ-CON	Pd NPs/TABE-PZ-CON/GCE	TEA	DNA/RNA recognition	microRNA-21	100 aM~1 nM	17.9 aM	extracts of cancer cells (HeLa, MCF-7)	[46]
	Ru-MCOF	HT/H2-Fc/AuNPs/Ru-MCOF/GCE	TPrA	DNA/RNA recognition	microRNA-155	10 aM~1 nM	3.02 aM	cancer cells (MCF-7 and HeLa)	[40]
	ET-COF-COOH	AIE-COF/CuS@Ag SC	DEDA	Catalyzed hairpin assembly strategy	miRNA-124-3p	1 fM~10 nM	0.49 fM	glioma tumor	[67]
	CTF	rHp/Cu_x_Mn_3_-x(HITP)_2_/ssDNA/CTF/AE	K_2_S_2_O_8_	Aptamer recognition	carcinoembryonic antigen (CEA)	1 pg/mL~50 ng/mL	2.91 fg/mL	human serum	[68]
	HHTP-HATP-COF	S_2_O_8_^2–^/HHTP-HATP-COF/GCE	K_2_S_2_O_8_	Pre-reduction+ aptamer recognition	thrombin (TB)	100 aM~1 nM	62.1 aM	diluted human serum	[35]
	TCPB-DMTA-COF	CoOOH/TCPB-DMTA-COF/GCE	K_2_S_2_O_8_	Enzyme/nano-enzyme catalysis	alkaline phosphatase (ALP)	0.01~100 U/L	6.0 × 10^−3^ U/L	human serums	[69]
	Ru-MCOFs	Ru-MCOFs/GCE	TEA	DNAzymes catalysis	cardiac troponin I (cTnI)	1 fg/mL~10 ng/mL	0.42 fg/mL	human serum	[70]
	TFPPy-DMeTHz-COF	TFPPy-DMeTHz-COF/GCE	/	catalytically decreased pH	glucose	0.1~500 μM	0.031 μM	human serum	[41]
	1D-COF	1D-COF/GCE	TPrA	Electro-oxidization and ECL RET	dopamine (DA)	0.1~1000 nM	4.05 pM	10% normal human serum	[55]
	T-COF	T-COF/Electrode	TEA with Ag^+^ as co-reactant accelerator	Immune recognition reaction	thymic stromal lymphopoietin (TSLP)	1.00 pg/mL~4.00 ng/mL	2.72 pg/mL	serum	[65]
	TC-COF	TC-COF/GCE	TEA with Au@Rh as co-reactant accelerator	Immune recognition reaction	adeno-associated virus serotype 8 (AAV8)	10^8^~5 × 10^11^ vg/mL	10^7.15^ vg/mL	serum	[66]
	PTCA-COF	PTCA-COF/Cu-HHTP/GCE	K_2_S_2_O_8_	Specific adsorption	diclazepam	0.1 pg/L~10 ng/L	26 fg/L	bear	[63]
	PTCA-COF	Fc-aptamer/Ag@CuCo_2_O_4_/PTCA-COF/GCE	K_2_S_2_O_8_	Aptamer recognition	cathinones 4-chloroethcathinone (4-CEC)	1 pg/L~1 μg/L	0.25 pg/L	e-Cigarette	[71]
	A-COFs	Tb@A-COF/Ag NWs	TPrA	Aptamer recognition	isobutyryl fentanyl (iBF)	1 fg/L~100 ng/L	0.897 fg/L	beer beverages	[62]
COF-supported ECL systems	cCTF	asDNA/MBs-Py-Ru-cCTFs/GCE	TPrA	DNA/RNA recognition	miRNA-182	1~100 fM	0.28 fM	human serum	[37]
	TAPP-COF	PtNPs/Eu@TAPP-COF/GCE	K_2_S_2_O_8_	DNA/RNA recognition	miRNA-21	100 aM~100 pM	21 aM	human serum	[56]
	TBA-TAPB	WS_2_@COF Lu CDs/GCE	H_2_O_2_	DNA/RNA recognition	miR-126	/	30.61 aM	/	[64]
	Tp-TAPB COF	N-dots@COF	H_2_O_2_	DNA/RNA recognition	miRNA-381	1 fM~10 nM	0.13 fM	ascites	[72]
	COF-LZU1	Ru@COF-LZU1@γ-CD-MOF-Au-Ab2	DBAE	Immune recognition reaction	CYFRA 21-1	10 fg/mL~50 ng/mL	5.5 fg/mL	human serum	[73]
	COF-LZU1	Au@COF-LZU1@Ru/GCE	TPrA	Aptamer recognition	Rare circulating tumor cells (CTCs)	8~100,000 cells/mL	2 cells/mL	human peripheral blood	[74]
	COF-LZU1	ABEI-COFs/GCE	TPrA	Aptamer recognition	cytochrome c	1 fg/mL~0.1 ng/mL	0.73 fg mL^−1^	human serum	[75]
	TPB-DVA COF	Ir(ppy)_3_ and Ru(bpy)_3_^2+^/TPB-DVA COF	TPrA	Immune recognition reaction	amyloid-β (Aβ)	0~50 pM	1 pM	100-fold diluted serum	[76]
	Py-PB-COF	Au@COF/Fc/GCE	O_2_	Aptamer recognition	acetylcholinesterase (AChE)	0.5 nM~1 μM	0.17 nM	serum	[77]
	CTpBD	MIP/UCNPs/CTpBD-Au/GCE	K_2_S_2_O_8_	Molecularly imprinting recognition	dopamine	0.01 pM~1 μM	2 fM	rat blood serum	[9]
	NH_2_-COFs	RuP/Pd NPs@COFs/GCE	TPrA	Aptamer recognition	progesterone (P4)	0.80 pM~7.95 μM	0.45 pM	human serum	[78]

**Table 2:** Summary on the COF-based ECL lumiphores and COF-supported ECL systems for food safety assay.

App.	COF	ECL System	Co-Reactant	Sensing Mechanism	Analytes	Linear Range	LOD	Samples	Ref.
COF-based ECL lumiphores	COF-AI-ECL	CAP-MIP/COF-AI-ECL/Co_3_O_4_/Au	H_2_O_2_	Molecularly imprinting recognition	chloramphenicol (CAP)	0.5~400 pM	0.118 pM	honey, milk, chicken	[18]
	COF-AIECL	MIP/COF-AIECL/Fe3O4@Pt NPs/GCE	H_2_O_2_	Molecularly imprinting recognition	ciprofloxacin (CFX)	2 pM~3 nM	0.598 pM	milk	[79]
	TP-ML COF	TP-ML COF/GCE	K_2_S_2_O_8_	Aptamer recognition	Malathion (Mal)	0.001~100 ng/mL	19.03 fg/mL	/	[32]
	TFPPy-TPh-COF	ZIF-8/TFPPy-TPh COF/GCE	TPrA	Specific recognition of AChE enzyme	Malathion (Mal)	0.01~1000 ng/mL	2.44 pg/mL	apple, lettuce, pak choi	[42]
	PTCA-COF	Fc-DNA/Au NPs/PTCA-COF/GCE	K_2_S_2_O_8_	DNA/RNA recognition	acetamiprid	0.1 nM~0.1 mM	2.7 pM	lettuce sample solutions	[43]
	DAFB-DCTP	MIPs/DAFB-DCTP @CNNs/GCE	K_2_S_2_O_8_	Molecularly imprinting recognition	carbaryl	0.1 nM~0.5 mM	46.7 pM	milk powder, fruit wine	[80]
	TFPT-TAPB-COF	TFPT-TAPB-COF/GCE	K_2_S_2_O_8_	Immune recognition reaction	zearalenone (XEN)	10 fg/mL~100 ng/mL	7.9 fg/mL	corn, wheat flour, tea	[81]
COF-supported ECL systems	COF-LZU1	Ru@COF-LZU1/GCE	TPrA	Aptamer recognition	aflatoxin M1 (AFM1)	0.03 pg/mL~0.3 mg/mL	0.009 pg/mL	defatted milk	[21]
	COF-LZU1	DTS/Au/COF@Ru/GCE	TPrA	Aptamer recognition	ochratoxin A (OTA)	10 fg/mL~100 ng/mL	3.5 fg/mL	peanut, corn and wine	[82]
	COF-LZU1	Ru@COF-LZU1-MBs	TPrA	Aptamer recognition	*N*-nitrosodimethylamine (NDMA)	100 fg/mL~100 ng/mL	9.11 fg/mL	seafood	[83]
	COF-LZU1	COF/CsPbBr_3_/ITO	ascorbic acid	Aptamer recognition	T-2 toxin	10 fg/mL~100 ng/mL	3.56 fg/mL	maize	[84]
	COF-300	MIPs/CsPbBr_3_/COF-300-Au/GCE	K_2_S_2_O_8_	Molecularly imprinting recognition	benzo(a)pyrene (BaP)	10 fM~10 μM	4.1 fM	edible oils	[85]
	Zr-amide-Por-based 2D COF	Tc-MIP/COF/GCE	luminol-H_2_O_2_	Molecularly imprinting recognition	tetracycline (Tc)	5~60 pM	2.3 pM	milk	[86]
	TAPB-DMTP	Ru@SiO_2_-CMIPs/GCE	TPrA	Molecularly imprinting recognition	cyanidin-3-O-glucoside (C3G)	0.0025~50 ng/mL	0.15 pg/mL	Blueberry, mulberry	[87]

**Table 3:** Summary on the COF-based ECL systems for environmental monitoring.

Entry	COF	ECL System	Co-Reactant	Sensing Mechanism	Analytes	Linear Range	LOD	Samples	Ref.
1	TP-TBDA	TP-TBDA@TCNQ/GCE	Na_2_S_2_O_8_	Charge-transfer	uranyl ions (UO_2_^2+^)	10~5000 nM	3 nM	waste water	[59]
2	BCBA–TBTN-AO	BCBA–TBTN-AO/GCE	O_2_	Chelating and electron transfer	uranyl ions (UO_2_^2+^)	0.001~1000 nM	0.36 pM	/	[47]
3	BTT-TBTN	BTT-TBTN-AO/GCE	O_2_	Molecularly imprinting recognition	uranyl ions (UO_2_^2+^)	0~5 μM	3.5 pM	Seawater/freshwater	[49]
4	TBTN-TFPT	TBTN-TFPT/GCE	K_2_S_2_O_8_	Stronger affinity	lutetium ion (Lu^3+^)	0.005~20 μM	1.6 nM	/	[50]
5	DVA-COF	DVA-COF/GCE	TPrA	Charge transfer	I^−^ and iodixanol	0.01~500 μM	39 pM for I^−^; 1.15 nM for iodixanol	lake water	[54]
6	TFPB-BD(OMe)_2_-H	TFPB-BD(OMe)_2_-H/GCE	K_2_S_2_O_8_	Strong oxidation and electron affinity	As(V) ions	0.001~5 μM	0.33 nM	lake water	[33]
7	RuCOF	RuCOF/Au electrode	K_2_S_2_O_8_	Selective N, N′-chelating sites	Hg^2+^ and Zn^2+^	1 nM~1μM	4.71 nM for Hg^2+^; 6.57 nM for Zn^2+^	real water	[88]
8	TAPB-DVA COF	AuNCs@COFs/GCE	TEA	Aptamer recognition	Pb^2+^	10 pM~5 μM	7.9 pM	soil	[60]
9	PTCA-COF	AuNCs/PTCA-COF/GCE	TEA	Aptamer recognition	Cd^2+^	1 pM~5 nM	0.66 pM	river water	[61]
10	TDCOF	HCNSFe_3_O_4_@ TDCOF	O_2_	Tc suppressed the charge transfer	tetracycline (Tc)	0.10~10 μg/L	0.031 μg/L	/	[89]
11	TAPB-DMTP-COF	ZnCdS@COF/GCE	K_2_S_2_O_8_	Aptamer recognition	oxytetracycline (OTC)	1 pg/mL~1 μg/mL	0.287 pg/mL	lake water	[90]
12	NC-COF_Py-Bpy_	NC-COF_Py-Bpy_/GCE	TPrA	ECL resonance energy transfer	doxorubicin (DOX)	0.01~100 μM	0.24 nM	/	[51]
13	CD-COF	CD-COF/S_2_O_8_^2−^/Bu_4_N^+^	K_2_S_2_O_8_ with Bu_4_NPF_6_ as co-reactant accelerator	Aptamer recognition	bisphenol A (BPA)	0.01 pM~10 μM	2.21 fM	river water, bottled water, tap water	[91]

**Table 4:** Summary on the COF-based ECL systems for enantioselective sensing.

Entry	COF	ECL System	Co-Reactant	Sensing Mechanism	Analytes	Linear Range	LOD	Samples	Ref.
1	(*R*)-PTCDA-RMP	(*R*)-PTCDA-RMP/GCE	K_2_S_2_O_8_	Chiral ECL-active unit	L-penicillamine (L-PA)	50 μM~1 mM,	9.74 μM	human urine	[92]
2	triPhPy^+^-(*S*)-CHA	triPhPy^+^-(*S*)-CHA/GCE	K_2_S_2_O_8_	Chiral ECL-active unit	D-Arg	/	/	/	[93]
3	aminal-linked COF	β-CD/aminal-linked COF/GCE	TPrA	Competitive host-guest interaction of β-CD	L-phenylalanine (L-Phe)	0.05~100 μM	0.045 μM	human serums	[36]
4	Bpy-COF	Ru(bpy)(*S*-CHDA)_2_^2+^/GCE	K_2_S_2_O_8_	Chiral ECL-active unit	L-Trp	10 μM~0.1 mM	/	/	[94]
5	Ph-triPy^+^-(R)-Ru(II)	Ph-triPy^+^-(R)-Ru(II)/GCE	Na_2_S_2_O_8_	Chiral ECL-active unit	D-Leu/L-Leu-OH	/	/	/	[57]
6	TriPhPy^+^-BiPh	TriPhPy+@(*R*/*S*)-CHA	K_2_S_2_O_8_	Chiral ECL-active unit	(*R*)/(*S*)-amino alcohol	/	/	/	[95]

## Data Availability

Not applicable.

## Abbreviations

The following abbreviations are used in this manuscript:

4-CEC	cathinones 4-chloroethcathinone
AAV8	adeno-associated virus serotype 8
ABEI	*N*-(4-aminobutyl)-*N*-ethylisoluminol
AChE	acetylcholinesterase
ACQ	aggregation-caused quenching
AD	Alzheimer’s disease
AFM1	aflatoxin M1
AIE	aggregation-induced emission
AIECL	aggregation-induced electrochemiluminescence
ALP	alkaline phosphatase
Aβ	amyloid-β
BAP	benzaldehyde-4,4′4″-phosphinidynetris
BAP	benzaldehyde,4,4′4″-phosphinidynetris
BaP	benzo(a)pyrene
BCBA	4-[4-[4-(4formylphenyl)-N-[4-(4-formylphenyl)phenyl]anilino]phenyl]benzaldehyde
BTA	benzene-1,2,4,5-tetramine
BTT	1,3,5-tris(4-formylphenyl)benzothiadiazole
C3G	cyanidin-3-O-glucoside
CAP	chloramphenicol
CB	conductance bands
CEA	carcinoembryonic antigen
CFX	ciprofloxacin
COF	covalent organic framework
CRT-ECL	covalent rigidification-triggered Electrochemiluminescence
CTCs	Rare circulating tumor cells
CTF	covalent triazine framework
cTnl	cardiac troponin I
cyt c	cytochrome c
DA	dopamine
D-A	donor–acceptor
DAFB	4-[4-[3,5-bis[4-(4-formylphenyl)phenyl]phenyl]phenyl]benzoic acid
D-Arg	D-arginine
DBAE	N,N’-dibutyl-2-hydroxyethylamine
DCTP	2,4,6-trimethylpyridine-3,5-dicarbonitrile
DEDA	N,N’-diethyl ethylenediamine
DFT	density function theory
DMeTHz	2,5-dimethoxyterephthalohydrazide
DMTP	2,5-dimethoxyterephthalaldehyde
DVA	2,5-divinylterephthalaldehyde
ECL	electrochemiluminescence
ESP	electrostatic-potential polarity
ET	electron transfer
ETB	4,4′,4″,4‴-(ethene-1,1,2,2-tetrayl)tetrabenzaldehyde
FIECL	framework-induced electrochemiluminescence
GCE	glassy carbon electrode
HATP	2,3,6,7,10,11-hexaaminotriphenylene
HCR	hybridization chain reaction
HHTP	2,3,6,7,10,11-hexahydroxytriphenylene
HOMOs	highest occupied molecular orbitals
iBF	isobutyryl fentanyl
ICT	intramolecular charge transfer
IRCT	intramolecular charge transfer
LOD	limits of detection
L-PA	L-penicillamine
L-Phe	L-phenylalanine
LUMOs	lowest unoccupied molecular orbitals
MA	melamine
Mal	Malathion
MCOF	metal covalent organic framework
MOF	metal–organic framework
NDMA	*N*-nitrosodimethylamine
OTA	ochratoxin A
PDA	terephthalaldehyde
PET	photoinduced electron
PTCDA	perylene-3,4,9,10-tetracarboxylic dianhydride
PZ	piperazine
RET	resonance energy transfer
Ru(dcbpy)_3_^2+^	tris(4,4′-dicarboxylicacid-2,2′-bipyridyl)ruthenium(II)
TABE	tetra-(4-aldehyde-(1,1-biphenyl))ethylene
TAPB	1,3,5-tris(4- aminophenyl)benzene
TB	thrombin
TBDA	2,5-di(thiophen-2-yl)benzene-1,4-diamine
TBTN	2,4,6-trimethylbenzene-1,3,5-tricarbonitrile
Tc	tetracycline
TCNQ	tetracyanoquinodimethane
TEA	triethylamine
TFPB	1,3,5-tri(4-formylphenyl)benzene
TFPD	4,40,40′-(pyrimidine-2,4,6-triyl) tribenzaldehyde
TFPP	4,40,40′-(pyridine-2,4,6-triyl) tribenzaldehyde
TFPPy	1,3,6,8-tetrakis(4-formylphenyl)pyrene
TFPT	4,40,40′-(1,3,5-triazine-2,4,6-triyl) tribenzaldehyde
TMT	trimethyltriazine/2,4,6-trimethyl-1,3,5-triazine
Tp	2,4,6-trihydroxybenzene-1,3,5-tricarbaldehyde
TPA	tris(4-formylphenyl)amine
TPh	(1,1′:3′,1″-terphenyl)-4,4″-diamine
TPrA	Tri-n-propylamine
TSLP	thymic stromal lymphopoietin
VB	valence bands
XEN	zearalenone

## References

[1] Miao W. (2008). Electrogenerated Chemiluminescence and Its Biorelated Applications. Chem. Rev..

[2] Liu Z., Qi W., Xu G. (2015). Recent Advances in Electrochemiluminescence. Chem. Soc. Rev..

[3] Ma X., Gao W., Du F., Yuan F., Yu J., Guan Y., Sojic N., Xu G. (2021). Rational Design of Electrochemiluminescent Devices. Acc. Chem. Res..

[4] Liu X., Zhao S., Tan L., Tan Y., Wang Y., Ye Z., Hou C., Xu Y., Liu S., Wang G. (2022). Frontier and Hot Topics in Electrochemiluminescence Sensing Technology Based on CiteSpace Bibliometric Analysis. Biosens. Bioelectron..

[5] Feng Y., Wang N., Ju H. (2022). Electrochemiluminescence Biosensing and Bioimaging with Nanomaterials as Emitters. Sci. China Chem..

[6] Giagu G., Fracassa A., Fiorani A., Villani E., Paolucci F., Valenti G., Zanut A. (2024). From Theory to Practice: Understanding the Challenges in the Implementation of Electrogenerated Chemiluminescence for Analytical Applications. Microchim. Acta.

[7] Dong Z., Du F., Zhang W., Tian Y., Xu G. (2025). Recent Advances in Tetraphenylethylene-Based Aggregation-Induced Electrochemiluminescence for Biosensing Applications. Curr. Opin. Electrochem..

[8] Sun Q., Ning Z., Yang E., Yin F., Wu G., Zhang Y., Shen Y. (2023). Ligand—Induced Assembly of Copper Nanoclusters with Enhanced Electrochemical Excitation and Radiative Transition for Electrochemiluminescence. Angew. Chem. Int. Ed..

[9] Gu Y., Wang J., Shi H., Pan M., Liu B., Fang G., Wang S. (2019). Electrochemiluminescence Sensor Based on Upconversion Nanoparticles and Oligoaniline-Crosslinked Gold Nanoparticles Imprinting Recognition Sites for the Determination of Dopamine. Biosens. Bioelectron..

[10] Cao Y., Zhu W., Li L., Zhang Z., Chen Z., Lin Y., Zhu J.-J. (2020). Size-Selected and Surface-Passivated CsPbBr3 Perovskite Nanocrystals for Self-Enhanced Electrochemiluminescence in Aqueous Media. Nanoscale.

[11] Lv W., Yang Q., Li Q., Li H., Li F. (2020). Quaternary Ammonium Salt-Functionalized Tetraphenylethene Derivative Boosts Electrochemiluminescence for Highly Sensitive Aqueous-Phase Biosensing. Anal. Chem..

[12] Han T., Cao Y., Chen H.-Y., Zhu J.-J. (2021). Versatile Porous Nanomaterials for Electrochemiluminescence Biosensing: Recent Advances and Future Perspective. J. Electroanal. Chem..

[13] Li C., Yang J., Xu R., Wang H., Zhang Y., Wei Q. (2022). Progress and Prospects of Electrochemiluminescence Biosensors Based on Porous Nanomaterials. Biosensors.

[14] Fu H., Xu Z., Hou H., Luo R., Ju H., Lei J. (2023). Framework-Enhanced Electrochemiluminescence in Biosensing. Chemosensors.

[15] Yang Z.-W., Li J.-J., Wang Y.-H., Gao F.-H., Su J.-L., Liu Y., Wang H.-S., Ding Y. (2023). Metal/Covalent-Organic Framework-Based Biosensors for Nucleic Acid Detection. Coord. Chem. Rev..

[16] Cao Y., Wu R., Gao Y.-Y., Zhou Y., Zhu J.-J. (2024). Advances of Electrochemical and Electrochemiluminescent Sensors Based on Covalent Organic Frameworks. Nano-Micro Lett..

[17] Li Y.-J., Cui W.-R., Jiang Q.-Q., Wu Q., Liang R.-P., Luo Q.-X., Qiu J.-D. (2021). A General Design Approach toward Covalent Organic Frameworks for Highly Efficient Electrochemiluminescence. Nat. Commun..

[18] Li S., Ma X., Pang C., Wang M., Yin G., Xu Z., Li J., Luo J. (2021). Novel Chloramphenicol Sensor Based on Aggregation-Induced Electrochemiluminescence and Nanozyme Amplification. Biosens. Bioelectron..

[19] Wei W., Ze H., Qiu Z. (2023). Reticular Sensing Materials with Aggregation-Induced Emission Characteristics. TrAC Trends Anal. Chem..

[20] Luo R., Lv H., Liao Q., Wang N., Yang J., Li Y., Xi K., Wu X., Ju H., Lei J. (2021). Intrareticular Charge Transfer Regulated Electrochemiluminescence of Donor–Acceptor Covalent Organic Frameworks. Nat. Commun..

[21] Zeng W.-J., Wang K., Liang W.-B., Chai Y.-Q., Yuan R., Zhuo Y. (2020). Covalent Organic Frameworks as Micro-Reactors: Confinement-Enhanced Electrochemiluminescence. Chem. Sci..

[22] Ni Y., Jiang D., An X., Wang W., Xu F., Liu H.W., Chen Z. (2024). Low-Triggering-Potential Electrochemiluminescence Based on Mental-Organic Frameworks Encapsulation of Ruthenium for Synthetic Cathinone Detection by Coupling Photonic Crystal Light-Scattering Signal Amplification of Covalent-Organic Frameworks. Anal. Chim. Acta.

[23] Qin X., Zhan Z., Ding Z. (2023). Progress in Electrochemiluminescence Biosensors Based on Organic Framework Emitters. Curr. Opin. Electrochem..

[24] Zhu J., Wen W., Tian Z., Zhang X., Wang S. (2023). Covalent Organic Framework: A State-of-the-Art Review of Electrochemical Sensing Applications. Talanta.

[25] Wei Y., Yang W., Guo H. (2023). Functional COFs for Electrochemical Sensing: From Design Principles to Analytical Applications. Chem. Select..

[26] Fan J., Jiang G., Li J., Qi J., Nalumansi H.S., Wang J., Pi F. (2025). Covalent Organic Frameworks (COFs)-Based Advanced Sensors for Detecting Food Contaminants: Design Strategies and Applications. Microchem. J..

[27] Luo R., Zhu D., Ju H., Lei J. (2023). Reticular Electrochemiluminescence Nanoemitters: Structural Design and Enhancement Mechanism. Acc. Chem. Res..

[28] Yang L., Li J. (2023). Recent Advances in Electrochemiluminescence Emitters for Biosensing and Imaging of Protein Biomarkers. Chemosensors.

[29] Luo R., Luo X., Xu H., Wan S., Lv H., Zou B., Wang Y., Liu T., Wu C., Chen Q. (2024). Reticular Ratchets for Directing Electrochemiluminescence. J. Am. Chem. Soc..

[30] Hou S., Liu G., Gao H., Li H., Liang X. (2025). Highly Efficient Aggregation-Induced Electrochemiluminescence Performance of Covalent Organic Frameworks with Electron-Rich Conjugated Structures. Chem. Eur. J..

[31] Zanut A., Fiorani A., Canola S., Saito T., Ziebart N., Rapino S., Rebeccani S., Barbon A., Irie T., Josel H.-P. (2020). Insights into the Mechanism of Coreactant Electrochemiluminescence Facilitating Enhanced Bioanalytical Performance. Nat. Commun..

[32] Luo W., Li Z., Qiu H., Zhu Z., Li J., Hu Y., Lai X., Yin S., Tang J. (2025). Planar β Keto-Enamine-Based Covalent Organic Frameworks as New Emitters for Electrochemiluminescence Sensing of Organophosphate Pesticides. Biosens. Bioelectron..

[33] Mao X.-L., Luo Q.-X., Cai Y.-J., Liu X., Jiang Q.-Q., Zhang C.-R., Liang R.-P., Qiu J.-D. (2023). Structural Isomerism of Covalent Organic Frameworks Causing Different Electrochemiluminescence Effects and Its Application for the Detection of Arsenic. Anal. Chem..

[34] Xu H., Luo R., Lv H., Liu T., Liao Q., Wang Y., Zhong Z., Wu X., Lei J., Xi K. (2025). Deciphering a Volcano-Shaped Relationship between Radical Stability and Reticular Electrochemiluminescence. Nat. Commun..

[35] Zhang J.-L., Yao L.-Y., Yang Y., Liang W.-B., Yuan R., Xiao D.-R. (2022). Conductive Covalent Organic Frameworks with Conductivity- and Pre-Reduction-Enhanced Electrochemiluminescence for Ultrasensitive Biosensor Construction. Anal. Chem..

[36] Song L., Gao W., Wang S., Bi H., Deng S., Cui L., Zhang C.-Y. (2022). Construction of an Aminal-Linked Covalent Organic Framework-Based Electrochemiluminescent Sensor for Enantioselective Sensing Phenylalanine. Sens. Actuators B-Chem..

[37] Bao J.-Y., Liu W., Chen C., Zhu H.-T., Wang A.-J., Yuan P.-X., Feng J.-J. (2024). Automated ECL Aptasensing Platform from an Intrarticular Radical Annihilation Route for Distinguishing Glioma Stages. Anal. Chem..

[38] Wang J., Yu Y., Yu H., Wang W., Shen L.-L., Zhang G.-R., Mei D. (2023). Covalent Triazine Framework Encapsulated Ultrafine PdAu Alloy Nanoclusters as Additive-Free Catalysts for Efficient Hydrogen Production from Formic Acid. ACS Catal..

[39] Zhang R., Cai W., Yuan S., Zhao L., Wang L., Li J., Wu D., Kong Y. (2024). Ionic Covalent-Organic Frameworks Composed of Anthryl-Extended Viologen as a Kind of Electrochemiluminescence Luminophore. ACS Appl. Mater. Interfaces.

[40] Yang Y., Jiang H., Li J., Zhang J., Gao S.-Z., Lu M.-L., Zhang X.-Y., Liang W., Zou X., Yuan R. (2023). Highly Stable Ru-Complex-Based Metal-Covalent Organic Frameworks as Novel Type of Electrochemiluminescence Emitters for Ultrasensitive Biosensing. Mater. Horiz..

[41] Zuo M., Cui L., Wang S., Wei W., Gao W., Zhang C. (2023). Development of an Exogenous Coreactant-Free Electrochemiluminescent Sensor for Sensing Glucose. Analyst.

[42] Song L., Zhang Q., Min L., Guo X., Gao W., Cui L., Zhang C. (2024). Electrochemiluminescence Enhanced by Isolating ACQphores in Imine-Linked Covalent Organic Framework for Organophosphorus Pesticide Assay. Talanta.

[43] Li Y., Yang F., Yuan R., Zhong X., Zhuo Y. (2022). Electrochemiluminescence Covalent Organic Framework Coupling with CRISPR/Cas12a-Mediated Biosensor for Pesticide Residue Detection. Food Chem..

[44] Zhang J.-L., Yang Y., Liang W.-B., Yao L.-Y., Yuan R., Xiao D.-R. (2021). Highly Stable Covalent Organic Framework Nanosheets as a New Generation of Electrochemiluminescence Emitters for Ultrasensitive MicroRNA Detection. Anal. Chem..

[45] Luo Q.-X., Cui W.-R., Li Y.-J., Cai Y.-J., Mao X.-L., Liang R.-P., Qiu J.-D. (2021). Construction of Sp2 Carbon-Conjugated Covalent Organic Frameworks for Framework-Induced Electrochemiluminescence. ACS Appl. Electron. Mater..

[46] Zhang J.-L., Wang T.-T., Liang W.-B., Yuan R., Xiao D.-R. (2024). Rigidifying AIEgens in Covalent Organic Framework Nanosheets for Electrochemiluminescence Enhancement: TABE-PZ-CON as a Novel Emitter for microRNA-21 Detection. Anal. Chim. Acta.

[47] Cui W.-R., Li Y.-J., Jiang Q.-Q., Wu Q., Luo Q.-X., Zhang L., Liang R.-P., Qiu J.-D. (2021). Covalent Organic Frameworks as Advanced Uranyl Electrochemiluminescence Monitoring Platforms. Anal. Chem..

[48] Li Y.-J., Cui W.-R., Jiang Q.-Q., Liang R.-P., Li X.-J., Wu Q., Luo Q.-X., Liu J., Qiu J.-D. (2021). Arousing Electrochemiluminescence Out of Non-Electroluminescent Monomers within Covalent Organic Frameworks. ACS Appl. Mater. Interfaces.

[49] Cui W.-R., Li Y.-J., Jiang Q.-Q., Wu Q., Liang R.-P., Luo Q.-X., Zhang L., Liu J., Qiu J.-D. (2022). Tunable Covalent Organic Framework Electrochemiluminescence from Non-Electroluminescent Monomers. Cell Rep. Phys. Sci..

[50] Luo Q.-X., Cai Y.-J., Mao X.-L., Li Y.-J., Zhang C.-R., Liu X., Chen X.-R., Liang R.-P., Qiu J.-D. (2022). Tuned-Potential Covalent Organic Framework Electrochemiluminescence Platform for Lutetium Analysis. J. Electroanal. Chem..

[51] Cao X., Song L., Yang Y., Chu W., Zou X., Sun B., Yin H., Cui L. (2025). Remarkable Increase in Electrochemiluminescence of Isomeric Bipyridine-Based Covalent Organic Frameworks via Regulating the Direction of Imine Linkage for Sensing Application. J. Colloid Interface Sci..

[52] Liu T., Tao Q., Wang Y., Luo R., Ma J., Lei J. (2024). Tailored Cis-Trans Isomeric Metal-Covalent Organic Frameworks for Coordination Configuration-Dependent Electrochemiluminescence. J. Am. Chem. Soc..

[53] Hou H., Wu Y., Wan J., Luo R., Wu L., Zhao Y., Wu X., Lei J. (2025). P–π Conjugation-Promoted Electrochemiluminescence of Halogenated Covalent Organic Framework Nanoemitters. Angew. Chem. Int. Ed..

[54] Chu W., Cao X., Song L., Yang Y., Gao W., Cheng L., Ai S., He W., Cui L. (2025). Improving Electrochemiluminescence with Vinyl-Group-Anchored Covalent-Organic Frameworks for Detection of Iodide Ions and Iodixanol. Sens. Actuators B Chem..

[55] Song L., Gao W., Jiang S., Yang Y., Chu W., Cao X., Sun B., Cui L., Zhang C. (2024). One-Dimensional Covalent Organic Framework with Improved Charge Transfer for Enhanced Electrochemiluminescence. Nano Lett..

[56] Wang C., Xiong Z., Feng X., Xu S., Zhang Y., Zhang Y., Zeng L., Tan J., Ren Y. (2024). Novel Pore Confinement Enhanced Europium Functionalized Porphyrin Covalent Organic Framework Electrochemiluminescence Microreactor and Its Application to microRNA-21 Detection. Sens. Actuators B Chem..

[57] Tan L., Cai W., Wang F., Li J., Wu D., Kong Y. (2024). Postsynthetic Modification Strategy for Constructing Electrochemiluminescence-Active Chiral Covalent Organic Frameworks Performing Efficient Enantioselective Sensing. Anal. Chem..

[58] Meng X., Zheng L., Luo R., Kong W., Xu Z., Dong P., Ma J., Lei J. (2024). Bimodal Oxidation Electrochemiluminescence Mechanism of Coreactant-Embedded Covalent Organic Frameworks via Postsynthetic Modification. Angew. Chem. Int. Ed..

[59] Jiang Q.-Q., Li Y.-J., Wu Q., Wang X., Luo Q.-X., Mao X.-L., Cai Y.-J., Liu X., Liang R.-P., Qiu J.-D. (2023). Guest Molecular Assembly Strategy in Covalent Organic Frameworks for Electrochemiluminescence Sensing of Uranyl. Anal. Chem..

[60] Li L., Zhao W., Wang Y., Liu X., Jiang P., Luo L., Bi X., Meng X., Niu Q., Wu X. (2023). Gold Nanocluster-Confined Covalent Organic Frameworks as Bifunctional Probes for Electrochemiluminescence and Colorimetric Dual-Response Sensing of Pb2+. J. Hazard. Mater..

[61] Zhen M., Wang Y., He Y., Luo L., Ma G., Lv W., Li L., You T. (2025). “Kill Two Birds with One Stone” Role of PTCA-COF: Enhanced Electrochemiluminescence of Au Nanoclusters via Radiative Transitions and Electrochemical Excitation for Sensitive Detection of Cadmium Ions. Sens. Actuators B Chem..

[62] Fan B., Jiang D., Ni Y., Hu Z., Sun Z., Li H., Wang W., Chen Z. (2025). Synergistic Coupling of Antenna Effect and Schottky Junction in Tb-Doped Covalent Organic Framework for Enhanced Electrochemiluminescence Sensing of Isobutyryl Fentanyl. Anal. Chem..

[63] An X., Jiang D., Cao Q., Xu F., Shiigi H., Wang W., Chen Z. (2023). Highly Efficient Dual-Color Luminophores for Sensitive and Selective Detection of Diclazepam Based on MOF/COF Bi-Mesoporous Composites. ACS Sens..

[64] Han Y., Ren X., Wu T., Lei Li Y., Ma H., Ru Z., Jia Y., Feng Gao Z., Du Y., Wu D. (2025). Effective Enrichment of Free Radicals through Nanoconfinement Boosts Electrochemiluminescence of Carbon Dots Derived from Luminol. Angew. Chem. Int. Ed..

[65] Jia Y., Zhu M., Zhang X., Jia D., Tian T., Shi B., Ru Z., Ma H., Wan Y., Wei Q. (2024). Nanobody-Based Microfluidic Immunosensor Chip Using Tetraphenylethylene-Derived Covalent Organic Frameworks as Aggregation-Induced Electrochemiluminescence Emitters for the Detection of Thymic Stromal Lymphopoietin. Anal. Chem..

[66] Du Y., Li G., Yang Y., Song L., Cui L., Wan Y., Zhang C. (2025). High-Performance Electrochemiluminescence Imaging Immunosensor for Adeno-Associated Virus Serotype 8 Detection Based on Nanobody-Functionalized Covalent Organic Frameworks and Au@Rh Catalytic Amplification. ACS Sens..

[67] Li W., Liang Z., Wang P., Li Z., Ma Q. (2025). CuS@Ag Heterostructure-Based Surface Plasmonic Coupling Electrochemiluminescence Sensor for Glioma miRNA-124-3p Detection. Biosens. Bioelectron..

[68] Zhang S., Li Z., Wang Y., Guo C., Guo R., Guo Y., Zhang Z. (2025). Development of an Electrochemiluminescence Aptasensor Combining Covalent-Triazine Framework Emitter with Exonuclease III-Driven DNA Walker for Sensitive CEA Detection. Microchem. J..

[69] Cui L., Zhu C., Hu J., Meng X., Jiang M., Gao W., Wang X., Zhang C. (2023). Construction of a Dual-Mode Biosensor for Electrochemiluminescent and Electrochemical Sensing of Alkaline Phosphatase. Sens. Actuators B Chem..

[70] Tang S.-H., Qin L., Yang W.-G., Yuan R., Yang J., Li Y., Hu S.-S. (2025). Electrochemiluminescence Immunoassay of cTnI with Ruthenium-Based Metal Covalent Organic Framework and Dual DNAzymes Cascade Amplification Strategy. Chem. Eur. J..

[71] An X., Jiang D., Ni Y., Wang W., Zhu Q., Xu F., Shiigi H., Chen Z. (2023). Synergistic Multieffect Catalytic Amplified Cathodic Electrochemiluminescence Biosensor via Target Binding-Induced Aptamer Conformational Changes for the Ultrasensitive Detection of Synthetic Cathinone. ACS Appl. Mater. Interfaces.

[72] Guo Y., Wang J., Ji F., Wang P., Ma Q. (2025). Natural Supramolecular Hydrogel/N-dots@COF Nanocomposite-Based ECL Sensor for Exosomal miRNA-381 Detection. Chem. Eng. J..

[73] Ren X., Shao M., Li X., Xie Z., Zhao J., Wang H., Gao M., Wu D., Ju H., Wei Q. (2024). Confinement-Enhanced Electrochemiluminescence by Ru(dcbpy)_3_^2+^-Functionalized γ-CD-MOF@COF-LZU1 Porous Hybrid Material as Micro-Reactor for CYFRA 21-1 Detection. Talanta.

[74] Guo M., Shen B., He W., Li X., Li X., Li M., Hu R., Zhang M., Yan Y. (2024). A Novel Electrochemiluminescent Cytosensor Using 3D Multivalent Aptamer Recognition and Covalent Organic Frameworks-Associated DNA Walker for Highly Efficient Capture and Detection of Rare CTCs. Chem. Eng. J..

[75] Shen L., Wang Y.-W., Shan H.-Y., Chen J., Wang A.-J., Liu W., Yuan P.-X., Feng J.-J. (2022). Covalent Organic Framework Linked with Amination Luminol Derivative as Enhanced ECL Luminophore for Ultrasensitive Analysis of Cytochrome c. Anal. Methods.

[76] Jia Y.-L., Xu C.-H., Li X.-Q., Chen H.-Y., Xu J.-J. (2023). Visual Analysis of Alzheimer Disease Biomarker via Low-Potential Driven Bipolar Electrode. Anal. Chim. Acta.

[77] Wang X., Wang J., Ding H., Dong Y. (2025). Detection of Acetylcholinesterase Based on ECL Resonance Energy Transfer between Luminol and Gold Nanoparticle Decorated Covalent Organic Framework. Microchem. J..

[78] Pan J.-J., Zhu H.-T., Chen J., Ma X.-Q., Wang A.-J., Yuan P.-X., Feng J.-J. (2024). The Dual ECL Signal Enhancement Strategy of Pd Nanoparticles Attached Covalent Organic Frameworks and Exonuclease Cycling Reaction for the Ultrasensitive Detection of Progesterone. Talanta.

[79] Li S., Pang C., Ma X., Wu Y., Wang M., Xu Z., Luo J. (2022). Aggregation-Induced Electrochemiluminescence and Molecularly Imprinted Polymer Based Sensor with Fe3O4@Pt Nanoparticle Amplification for Ultrasensitive Ciprofloxacin Detection. Microchem. J..

[80] Liu C., Cai L., Wang X., Guo Y., Fang G., Wang S. (2022). Construction of Molecularly Imprinted Sensor Based on Covalent Organic Frameworks DAFB-DCTP-Doped Carbon Nitride Nanosheets with High Electrochemiluminescence Activity for Sensitive Detection of Carbaryl. Microchem. J..

[81] Li Z., Wang Y., Gabrielli S., Cimarelli C., Guo C., Du M., Pellei M., Zhang Z. (2025). Donor–Acceptor Conjugated and Triazine-Containing Covalentorganic Framework: Construction of a Signal “on–off–on” Electrochemiluminescence Immunosensor for Efficiently Detecting Zearalenone. Sens. Actuators B Chem..

[82] Cao Y., Huang C., Li K., Zhang X., Tong M., Tu J., Yang K., Yuan S., Zhang H. (2025). CRISPR/Cas12a-Assisted Electrochemiluminescent Detection of Ochratoxin A Based on COF@Ru Coupled with a DNA Tetrahedral Scaffold. Anal. Methods.

[83] Zheng Z., Wang J., Ma M., Xu Y., Huang D., Wang J., Lin C., Lin Z., Luo F. (2025). Nanoconfined-Structured Ru@COF-LZU1 Microenvironment Facilitated Homogeneous Electrochemiluminescence Biosensor for Ultrasensitive N-Nitrosodimethylamine Detection. Sens. Actuators B Chem..

[84] Li J., Wu Z., Luo F., Lin Z., Wang J., Li R., Qiu B. (2024). Stable Halide Perovskite CsPbBr3 Nanocrystals Assisted by Covalent-Organic Frameworks for Electrochemiluminescence Analysis in an Aqueous Medium. Anal. Chem..

[85] Chi H., Wang L., Wang S., Liu G. (2023). An Electrochemiluminescence Sensor Based on CsPbBr3 -Zquantum Dots and Poly (3-Thiophene Acetic Acid) Cross-Linked Nanogold Imprinted Layer for the Determination of Benzo(a)Pyrene in Edible Oils. Food Chem..

[86] Ma X., Pang C., Li S., Xiong Y., Li J., Luo J., Yang Y. (2019). Synthesis of Zr-Coordinated Amide Porphyrin-Based Two-Dimensional Covalent Organic Framework at Liquid-Liquid Interface for Electrochemical Sensing of Tetracycline. Biosens. Bioelectron..

[87] Cui C., Fan Y., Chen Y., Wei R., Lv J., Yan M., Jiang D., Liu Z. (2024). Molecular Imprinting-Based Ru@SiO_2_-Embedded Covalent Organic Frameworks Composite for Electrochemiluminescence Detection of Cyanidin-3-O-Glucoside. Talanta.

[88] Hu H., Yin Z., Cui H., Xiong W., Yu F., Zhang J., Liao F., Wei G., Yang L., Zhang J. (2024). A Novel Dual-Detection Electrochemiluminescence Sensor for the Selective Detection of Hg2^+^ and Zn2^+^: Signal Suppression and Activation Mechanisms. Anal. Chim. Acta.

[89] Cao Y., Wu R., Zhou Y., Jiang D., Zhu W. (2022). A Bioinspired Photocatalysis and Electrochemiluminescence Scaffold for Simultaneous Degradation and In Situ Evaluation. Adv. Funct. Mater..

[90] Jiang K.-B., Wang L., Wei Y.-P., Chen J.-S., Liu X.-P., Mao C.-J., Jin B.-K. (2025). Electrochemiluminescent Aptasensor Based on Covalent Organic Framework and ZnCdS Composite for Sensitive Detection of Oxytetracycline. Sens. Actuators B Chem..

[91] Ma R., Jiang J., Ya Y., Lin Y., Zhou Y., Wu Y., Tan X., Huang K., Du F., Xu J. (2023). A Carbon Dot-Based Nanoscale Covalent Organic Framework as a New Emitter Combined with a CRISPR/Cas12a-Mediated Electrochemiluminescence Biosensor for Ultrasensitive Detection of Bisphenol A. Analyst.

[92] Ruan Y., Kuang X., Zheng X., Sun X., Kuang R. (2025). Bidirectional ECL Response of Chiral COFs for the Discrimination of Penicillamine Enantiomers. Talanta.

[93] Yuan S., Cai W., Zhao L., Wang L., Zhang R., Li J., Wu D., Kong Y. (2024). Strong Electrochemiluminescence Response Derived from Ionic Chiral Covalent Organic Frameworks for Enantioselective Discrimination of Amino Acid Enantiomers via an Electrostatic Attraction Effect. ACS Appl. Mater. Interfaces.

[94] Yuan S., Tan L., Zhao L., Wang F., Cai W., Li J., Wu D., Kong Y. (2024). Chiral Ru-Based Covalent Organic Frameworks as An Electrochemiluminescence-Active Platform for the Enantioselective Sensing of Amino Acids. ACS Appl. Mater. Interfaces.

[95] Zhao L., Cai W., Yuan S., Wang L., Zhang R., Li J., Wu D., Kong Y. (2024). Optically Pure Co(III) Complex Absorbed by Electrochemiluminescence-Active Covalent Organic Framework as an Enantioselective Recognition Platform to Give Opposite Responses Toward Amino Alcohol Enantiomers. ACS Appl. Mater. Interfaces.

[96] Hu C., Xie W., Liu J., Zhang Y., Sun Y., Cai Z., Lin Z. (2024). Bioinspired Iron Porphyrin Covalent Organic Frameworks-Based Nanozymes Sensor Array: Machine Learning-Assisted Identification and Detection of Thiols. ACS Appl. Mater. Interfaces.

[97] Yang P., Zhang H., Lai X., Wang K., Yang Q., Yu D. (2021). Accelerating the Selection of Covalent Organic Frameworks with Automated Machine Learning. ACS Omega.

[98] Wang H., Li Y., Xuan X., Wang K., Yao Y., Pan L. (2025). Machine Learning Accelerated Discovery of Covalent Organic Frameworks for Environmental and Energy Applications. Environ. Sci. Technol..

[99] Wang D., Lv H., Wan Y., Wu X., Yang J. (2023). Band-Edge Prediction of 2D Covalent Organic Frameworks from Molecular Precursor via Machine Learning. J. Phys. Chem. Lett..

